# A hybrid car-following behavior prediction framework for snowy weather based on stacking ensemble learning

**DOI:** 10.1371/journal.pone.0361759

**Published:** 2026-10-30

**Authors:** Zengjie Luo, Fan Geng, Zhaolei Zhang

**Affiliations:** 1 Changsha Technology Investment Management Co., Ltd., Changsha, China; 2 National Engineering Research Center of Highway Maintenance Technology, Changsha University of Science and Technology, Changsha, China; 3 School of Traffic and Transportation Engineering, Central South University, Changsha, China; National University of Singapore, SINGAPORE

## Abstract

To improve the accuracy and robustness of vehicle car-following behavior prediction on snowy highways, this paper proposes a hybrid prediction framework based on Stacking ensemble learning. The framework integrates a theoretically driven improved Intelligent Driver Model (IDM) with two state-of-the-art data-driven models—the Prediction-enhanced Intelligent Driver Model (P-IDM) and a Transformer-LSTM hybrid network—within a Stacking ensemble architecture. First, an Icy Condition Enhanced IDM (ICES-IDM) model is developed by introducing a road adhesion coefficient mapping function and a speed perception error coefficient to parameterize the classical IDM for snowy environments. Second, a P-IDM model is constructed by incorporating a self-attention-based preceding vehicle speed prediction module into the classical IDM framework to enhance responsiveness to sudden maneuvers of the leading vehicle. Third, a Transformer-LSTM dual-stream network (TL-Net) is designed to jointly predict car-following acceleration by parallel extraction of temporal attention features and recurrent neural network features. Within the Stacking framework, ICES-IDM and P-IDM serve as physics-driven base learners, TL-Net serves as a data-driven base learner, and XGBoost is adopted as the meta-learner to construct a hybrid acceleration prediction model. The model is trained and validated using naturalistic driving data collected in Norway—comprising a dataset of 311,908 observations that link vehicle measurements with weather, road surface, and lighting conditions, along with a five-month road record dataset from Narvik. Experimental results demonstrate that the proposed model achieves a root mean square error (RMSE) in acceleration prediction under snowy conditions that is approximately 28.1% lower than that of the standalone ICES-IDM model, 9.2% lower than that of the TL-Net model, and 4.2% lower than that of the conventional Stacking-IDM-LSTM framework. The model exhibits excellent generalization capability across varying snow intensity levels, providing theoretical support for vehicle car-following safety warning systems in snowy environments.

## 1. Introduction

Snowy weather is one of the most significant adverse weather conditions affecting highway traffic safety. Snow accumulation and ice formation on road surfaces lead to a substantial reduction in tire–road adhesion coefficient, increasing vehicle braking distances by 2–3 times compared to dry pavement, and causing a sharp rise in car-following crash risks [1]. Alenzi et al. [2] systematically reviewed the seasonal effects of hot and cold climates on driving behavior, finding that drivers’ time headways in snowy environments increase by 40%–60% relative to normal weather conditions. Asgarzadeh et al. [3] analyzed the impacts of weather, road surface, and lighting conditions on traffic crash severity, further confirming the systematic increase in driving risks under adverse weather. Han et al. [4] proposed a deep learning trajectory prediction method integrating turning maneuver recognition for connected and autonomous vehicles in adverse weather. Research based on Norwegian naturalistic driving data indicates that precipitation type, precipitation intensity, road surface conditions, and lighting conditions have significant interactive effects on microscopic driving behavior [5]. Statistics show that traffic crash rates on snowy roads are 4–8 times higher than those on dry pavement, with rear-end collisions in car-following situations accounting for over 40% of these crashes [6]. In this context, accurately predicting vehicle car-following behavior in snowy environments is of critical importance for enhancing the warning timeliness of Advanced Driver Assistance Systems (ADAS) and ensuring driving safety.

However, car-following behavior in snowy environments exhibits complex characteristics including strong nonlinearity, high uncertainty, and significant individual heterogeneity [7,8]. On one hand, the road adhesion coefficient dynamically varies with snow intensity, directly constraining vehicle dynamic response capabilities. On the other hand, drivers’ perceptual abilities deteriorate under snowy conditions, leading to “risk compensation” behavior in car-following decisions—drivers actively reduce speed and increase following distances. Nevertheless, such conservative strategies do not always effectively eliminate safety hazards [9]. Therefore, developing predictive models that can accurately characterize car-following behavior in snowy environments has become a research hotspot and challenge in this field.

Vehicle car-following behavior prediction methods mainly fall into three categories: theory-driven, data-driven, and hybrid modeling approaches. Zhang et al. [10] and Wang et al. [11] have provided comprehensive reviews of the development of car-following models from multidisciplinary and systematic perspectives, respectively.

Theory-driven models are represented by the Intelligent Driver Model (IDM), the Optimal Velocity Model (OVM), and the Full Velocity Difference (FVD) model [12]. IDM has become a mainstream model in microscopic traffic simulation due to its clear physical meaning of parameters and concise formulation [11]. However, classical IDM assumes good road adhesion conditions and unbiased driver perception, making it significantly inadequate when directly applied to snowy environments. To address this issue, Li et al. [1] mapped the road adhesion coefficient to the maximum acceleration and comfortable deceleration parameters of IDM and introduced a speed perception error coefficient, constructing a snow-adaptive IDM model while employing safety field theory for car-following risk assessment. Du et al. [8] further introduced snowy road surface state parameters into the IDM framework, analyzing the operating safety states of intelligent vehicles on snowy roads. Wang et al. [12] proposed an adaptive IDM car-following strategy considering asymptotic stability and damping characteristics. For low-visibility scenarios such as foggy conditions, Yadav [6] proposed an improved car-following model incorporating visibility and traffic jerk effects. Liang and Pang [13] studied the mixed car-following process under foggy conditions with multiple preceding vehicle information, proposing a variable time headway model. Chen and He [14] designed an adaptive control framework for connected and automated vehicles in foggy environments to reduce car-following risks and energy consumption. Furthermore, Medina et al. [15] conducted verification and calibration of driver behavior and car-following metrics in microscopic traffic simulation. Gao [16] systematically investigated the calibration of freeway car-following models under various rainfall intensities. Mehta et al. [17] constructed a lattice hydrodynamic car-following model for curved roads considering visibility effects. Redhu et al. [18] respectively studied the effects of driver attention and advance reaction time on car-following dynamics under overtaking scenarios. These studies demonstrate that parametric improvement of IDM under adverse weather conditions is an effective approach to enhancing model adaptability [19].

Data-driven models leverage the powerful fitting capabilities of deep learning to directly learn car-following behavior from trajectory data. LSTM networks, with their gating mechanisms capable of capturing long-term dependencies, have been widely applied to acceleration prediction and driving intention recognition. Bai et al. [20] proposed a memory-based deep learning method with attention mechanism for modeling differentiated car-following behavior under sunny and rainy conditions. In recent years, the Transformer architecture, with its advantages in sequence modeling through self-attention mechanisms, has gradually replaced traditional RNN architectures. Wu et al. [21] proposed the Prediction-enhanced Intelligent Driver Model (P-IDM), embedding a Transformer module within the IDM framework for short-term prediction of preceding vehicle speed, effectively expanding the system's stable region and accelerating disturbance attenuation. Fang et al. [22] proposed a dynamic transformation car-following model for predicting traffic flow oscillations. Xu et al. [23] proposed an improved longitudinal driving car-following system considering a safe time domain strategy. However, purely data-driven models are prone to prediction bias or overfitting under extreme snowy conditions due to sparse training data [6].

Hybrid modeling and ensemble learning aim to fuse the interpretability of physical models with the nonlinear fitting capability of data-driven models. Stacking ensemble learning, which combines multiple base learners to form a strong learner, has achieved remarkable success in tasks such as dangerous lane-changing behavior recognition. Du and Zhao [5] employed Stacking ensemble learning to identify risky lane-changing behavior on snowy and icy surfaces, achieving an accuracy of 98.33%. Chen et al. [24] proposed an explainable Stacking-based learning model for traffic forecasting. In car-following behavior modeling, Jiang et al. [25] proposed strategies for reducing rear-end collision risks of connected and automated vehicles under different weather conditions. Wang et al. [10] provided a systematic review of car-following models for both human-driven and autonomous vehicles. However, existing hybrid methods have not yet fully exploited the synergistic potential between physical models and data-driven models in snowy scenarios, and in-depth investigations into nonlinear fusion of multiple heterogeneous base learners remain insufficient.

Despite significant progress made by the academic community in modeling vehicle car-following behavior under snowy and adverse weather conditions, the above literature review reveals several critical limitations in current research. Existing snow-adaptive car-following models primarily focus on the physical mapping of road adhesion coefficients, while behavioral features such as speed perception bias and prolonged reaction time caused by visual impairment and psychological tension under snowy conditions are mostly approximated using simple global parameters, lacking refined modeling of individual differences [2,10]. Although Yadav and Redhu [19] considered driver attention effects in V2V environments, their work was not specifically validated for low-adhesion snowy scenarios. Current mainstream hybrid modeling methods predominantly employ linear weighting, Kalman filtering, or simple Stacking ensembles [26]. With the application of autonomous vehicles, conducting research on driving behaviors in extreme environments can ensure the safety of autonomous vehicles [27,28]. Real-time implementation of machine learning models in transportation systems has attracted increasing attention in recent years. For example, trajectory prediction methods for autonomous driving have been surveyed with a focus on computational efficiency and real-time constraints [29,30].

The above limitations provide clear entry points for this study. To address the issue of simplistic fusion approaches, this paper introduces three heterogeneous base learners (ICES-IDM, P-IDM, and TL-Net) within a Stacking ensemble framework and employs the XGBoost meta-learner to achieve nonlinear adaptive fusion. The main contributions of this study are as follows:

Building upon the classical IDM, this paper introduces a road adhesion coefficient mapping function and a speed perception error coefficient to construct a snow-adaptive IDM model, providing physically interpretable prior constraints for the ensemble framework. Drawing upon the prediction-response concept, this paper embeds a self-attention-based Transformer module for short-term prediction of preceding vehicle speed within the IDM framework, enhancing the model's responsiveness to sudden maneuvers of the leading vehicle. A parallel network architecture is designed to employ Transformer for extracting long-range dependencies and LSTM for capturing transient nonlinear characteristics, enabling data-driven acceleration prediction. With ICES-IDM, P-IDM, and TL-Net as base learners and XGBoost as the meta-learner, this framework achieves nonlinear fusion of physics-driven and data-driven approaches, and employs error–disagreement decomposition to explain the underlying mechanism of ensemble gains.

## 2. Method

This section elaborates on the construction process of the proposed stacking hybrid prediction model, and the overall framework is shown in Fig 1. First, in view of the specificity of snowy environments, the classical Intelligent Driver Model (IDM) is parametrically improved to obtain the ICES-IDM model. Second, to enhance the responsiveness to sudden maneuvers of the leading vehicle, a Transformer-based speed prediction module is embedded within the IDM framework to construct the P-IDM model. Third, a Transformer-LSTM dual-stream network, termed TL-Net, is designed for purely data-driven acceleration prediction. Finally, the above three heterogeneous models are employed as base learners and integrated via the Stacking strategy with the XGBoost meta-learner to yield the final acceleration prediction.

**Fig 1:**
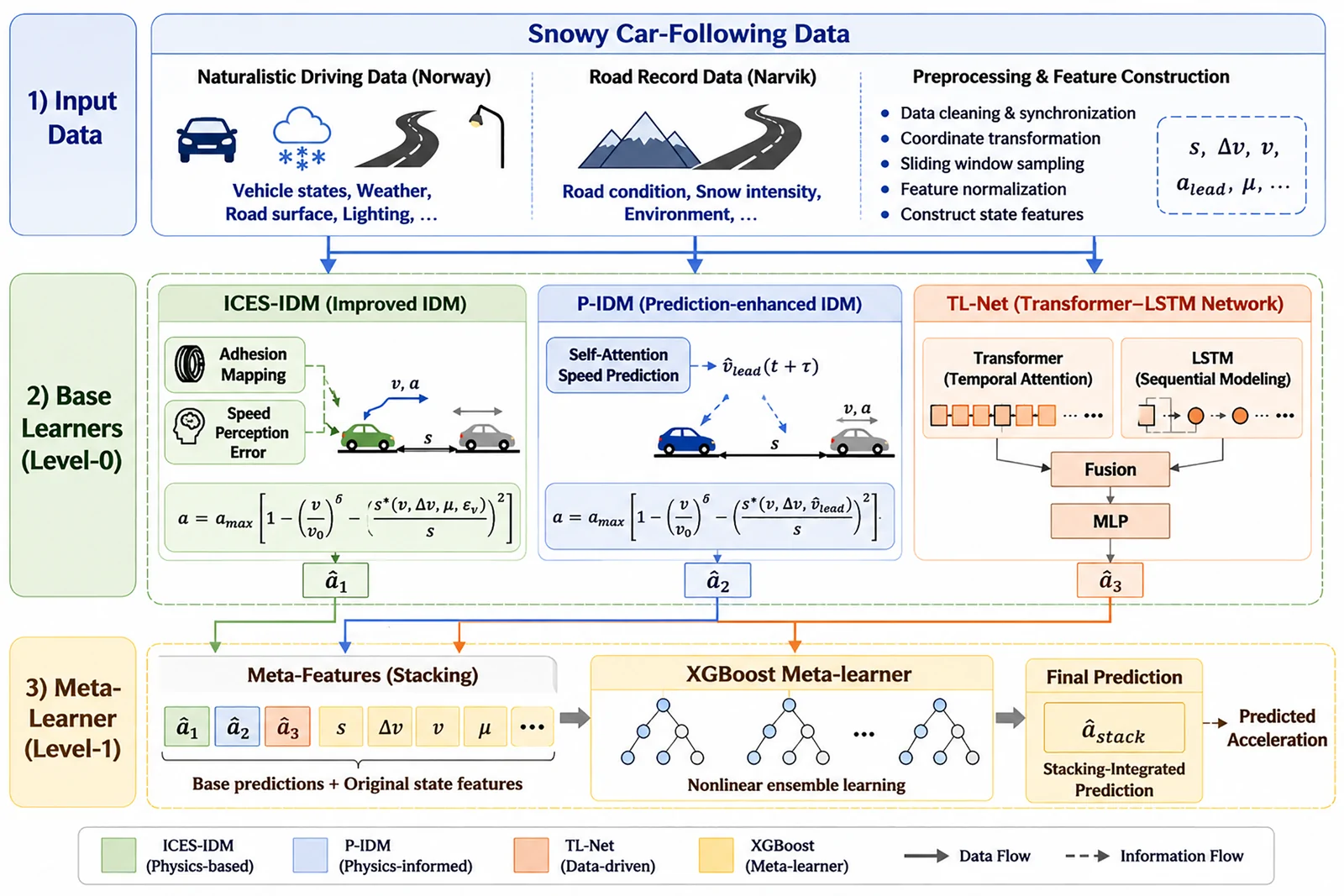
Stacking-Integrated car-following prediction method.

### 2.1. Icy Condition Enhanced IDM Model (ICES-IDM)

The Intelligent Driver Model (IDM) is a continuous car-following model proposed by Treiber et al. [11]. Owing to its clear physical meaning of parameters and concise formulation, it has been widely applied in microscopic traffic simulation and autonomous vehicle longitudinal control. The IDM assumes that a vehicle's acceleration is governed by two factors: the driver's tendency to reach the desired speed, and the need to maintain a safe distance from the preceding vehicle. Its acceleration control equation is given as:


$$\begin{array}{c} \dot{v}\left(t\right)=a\cdot \left[1-{\left(\frac{v\left(t\right)}{v_{\text{0}}}\right)}^{\delta }-{\left(\frac{s^{*}\left(v\left(t\right),\Delta v\left(t\right)\right)}{s\left(t\right)}\right)}^{\text{2}}\right] \end{array}$$ (1)

Where *v*(*t*) is the speed of the following vehicle at time *t*, $\dot{v}\left(t\right)$ is its acceleration, *s*(*t*)is the actual following distance, and Δ*v*(*t*)=*v*(*t*)−*v*_*lead*_(*t*) is the rela*t*ive speed be*t*ween *t*he following and preceding vehicles (negative values indicate closing). The parameter *a* denotes the maximum acceleration, *v*_0_ is the desired speed, and *δ* is the acceleration exponent controlling the rate of acceleration decay as the speed approaches the desired value. The desired minimum spacing s^*^ is computed as:


$$\begin{array}{c} s^{*}\left(v,\Delta v\right)=s_{\text{0}}+vT+\frac{v\Delta v}{2\sqrt{ab}} \end{array}$$ (2)

where *s*_0_ is the minimum safe spacing at standstill, *T* is the desired safe time headway, and *b* is the comfortable deceleration. In Eq. (2), the first term *s*_0_ ensures a physical spacing when stopped, the second term *vT* represents the distance covered in *T* seconds at speed *v*, and the third term $\frac{v\Delta v}{2\sqrt{ab}}$ is an additional safety margin to accommodate relative speed variations. When the following vehicle is faster than the leading vehicle (Δ*v* > 0), this term is positive, demanding a larger spacing; when the following vehicle is slower (Δ*v* < 0), this term is negative, allowing a moderately reduced spacing.

The classical IDM exhibits good descriptive capability on dry and well-paved roads. However, its parameters *a* and *b* are set as constants, failing to reflect the constraints imposed by reduced road adhesion coefficients on vehicle dynamic performance under snowy conditions. Moreover, the model assumes that the driver can precisely perceive the ego-vehicle speed, whereas in snowy environments, due to degraded visibility and covered lane markings, driver perception is often biased. To address these shortcomings, this paper parametrically improves the IDM for snowy environments.

The tire–road adhesion coefficient *μ* on snowy roads directly determines the maximum acceleration and maximum braking deceleration a vehicle can generate. Typically, the adhesion coefficient of dry asphalt pavement ranges from 0.7 to 0.8, while that on snowy roads can drop to 0.1–0.3. Drawing upon the exponential relationship between friction coefficient and acceleration capability in existing studies, this paper adopts an exponential mapping function with saturation characteristics:


$$\begin{array}{c} a\left(\mu \right)=a_{\text{0}}\cdot \left[\begin{array}{c} 1-e^{-\lambda_{a}(\mu -\mu_{min})} \end{array}\right] \end{array}$$ (3)

$$\begin{array}{c} b\left(\mu \right)=b_{\text{0}}\cdot \left[\begin{array}{c} 1-e^{-\lambda_{b}(\mu -\mu_{min})} \end{array}\right] \end{array}$$ (4)

where *a*_0_ = 3.5m/s^2^ and *b*_0_ = 3.0m/s^2^ are the standard maximum acceleration and comfortable deceleration on dry roads, respectively; *λ*_*a*_ and *λ*_*b*_ are decay coefficients controlling the rate of decline as the adhesion coefficient decreases (in this paper, *λ*_*a*_ = *λ*_*b*_ = 6.5); and *μ*_min_ = 0.1 is the lower bound of the adhesion coefficient, below which acceleration capability approaches zero when the road surface approaches pure ice. The rationality of this mapping function lies in the fact that on dry roads (*μ* ≈ 0.75), the exponential term*e*^−*λ*(0.75−0.1)^=*e*^−4.225^ ≈ 0.0147, hence *a*(*μ*)≈*a*_0_; on icy roads (*μ* = 0.18), the exponential term e^−6.5×0.08^ = e^−0.52^ ≈ 0.594, yielding *a*(*μ*)≈*a*_0_×(1 − 0.594)=1.42 m/s², which conforms to the physical law of degraded vehicle dynamic performance on snowy roads.

Under snowy conditions, due to lane markings being covered by snow, reduced environmental contrast, and whiteout phenomena, drivers’ visual perception is severely limited. To characterize such perceptual uncertainty, a perception error coefficient *ε* is defined, such that the perceived speed $\hat{v}$ and the actual speed *v*_real_ satisfy:


$$\begin{array}{c} \hat{v}=v_{\text{real}}\cdot (1+\varepsilon ) \end{array}$$ (5)

The value of *ε* typically ranges within [−0.3,0.3]. A positive value indicates overestimation of the ego-vehicle speed, leading the driver to adopt a more conservative following strategy by increasing the spacing; a negative value indicates underestimation, which may result in reduced following distances and delayed responses. Under snowy conditions, due to reduced external references, drivers tend to underestimate speed; therefore, *ε* is often positive.

Incorporating the perceived speed into the desired spacing calculation yields the modified desired spacing:


$$\begin{array}{c} s_{\varepsilon }^{*}\left(\hat{v},\Delta \hat{v}\right)=s_{\text{0}}+\hat{v}T+\frac{\hat{v}\Delta \hat{v}}{2\sqrt{a\left(\mu \right)b\left(\mu \right)}} \end{array}$$ (6)

where $\Delta \hat{v}$ also computed from the perceived speeds. Combining the above improvements, the acceleration control equation of the ICES-IDM model is:


$$\begin{array}{c} \dot{v}\left(t\right)=a\left(\mu )[1-{\left(\frac{\hat{v}\left(t\right)}{v_{\text{0}}}\right)}^{\delta }-{\left(\frac{s_{\varepsilon }^{*}\left(\hat{v}\left(t\right),\Delta \hat{v}\left(t\right)\right)}{s\left(t\right)}\right)}^{\text{2}}\right] \end{array}$$ (7)

Compared to the classical IDM, the ICES-IDM model can dynamically adjust the vehicle’s acceleration and braking capabilities according to the real-time adhesion coefficient *μ*, and simulate perceptual biases induced by snowy conditions through the perception error coefficient *ε*. Under extreme snowy conditions with sparse data, this model provides physically interpretable prior constraints and serves as a crucial base learner for the subsequent ensemble learning.

The exponential mapping with saturation is adopted because the available tire–road friction force does not decrease linearly with the adhesion coefficient; the marginal loss of available traction diminishes as the surface deteriorates, consistent with the friction–slip characteristics of tire–road interaction. Similar environmental parameterization strategies have been adopted in recent snowy car-following calibration studies [16], where the road friction coefficient is shown to directly modulate the acceleration and deceleration capabilities of IDM. The saturation form ensures that *a*(*u)* approaches zero as *μ* approaches *μ*_min_, preventing non-physical negative values at very low adhesion. With *λ*_*a*_ = *λ*_*b*_ = 6.5 and *μ*_min_ = 0.1, the normalized acceleration capability *a*(*u)*/*a*_0_ is approximately 0.28, 0.41, 0.48, 0.73, and 0.86 at *u* = 0.15,0.18,0.20,0.30, and 0.40, respectively. Thus, the mapping yields a steep reduction in effective acceleration capability in the very-low-adhesion regime and gradually saturates as adhesion improves. A sensitivity analysis with *λ* ∈ [4.0, 9.0] showed that the RMSE of ICES-IDM varies by less than 3.5%, indicating that the model is not critically sensitive to this parameter within a physically reasonable range. The perception error coefficient *ε* ∈ [−0.3, 0.3] is consistent with reported speed estimation errors of 10%–30% under degraded visibility. Positive values represent overestimation of the ego-vehicle speed and therefore induce a more conservative following response.

### 2.2. Prediction-enhanced intelligent driver model (P-IDM)

Both the classical IDM and its snow-improved version ICES-IDM belong to strictly reactive models, meaning that the acceleration of the ego vehicle depends only on the currently perceived state of the preceding vehicle (speed and spacing). Consequently, when the preceding vehicle performs emergency braking, the ego vehicle can only respond after detecting the speed reduction of the preceding vehicle, inevitably incurring a delay. On snowy roads, this delay can be amplified due to increased braking distances, leading to elevated rear-end collision risks. To alleviate this issue, this paper draws upon the “prediction-response” concept and constructs a Prediction-Enhanced Intelligent Driver Model (Predictive IDM, P-IDM). The core idea is to augment the IDM framework with a short-term speed prediction module for the preceding vehicle, enabling the ego vehicle to perceive the deceleration or acceleration intentions of the preceding vehicle in advance, thereby adjusting acceleration proactively.

The preceding vehicle speed prediction module adopts a Transformer encoder. The input is the historical speed sequence of the preceding vehicle over the pas *T*_*f*_ = 3 seconds (totaling 6 time steps at a sampling frequency of 2 Hz), and the output is the speed prediction for the future *T*_*p*_ = 1 second (2 time steps), denoted as ${\hat{v}}_{\text{lead}}(t+\Delta t)$. The Transformer encoder comprises 2 multi-head self-attention layers and 2 feedforward networks. Through the self-attention mechanism, it captures long-range dependency patterns in the speed sequence, effectively identifying the onset of speed reductions and predicting subsequent trends.

After obtaining the predicted speed of the preceding vehicle, it is substituted into the IDM desired spacing formula, replacing the original instantaneous speed *v*_lead_:


$$\begin{array}{c} s_{\text{P-IDM}}^{*}\left(v,\Delta v,{\hat{v}}_{\text{lead}}\right)=s_{\text{0}}+vT+\frac{v(v-{\hat{v}}_{\text{lead}})}{2\sqrt{ab}} \end{array}$$ (8)

The acceleration control equation of the P-IDM model takes the same form as the classical IDM, except that Eq. (2) is replaced by Eq. (8). When the predicted preceding vehicle speed v^lead*v*^lead is lower than the current ego vehicle speed *v*, the third term in Eq. (8) becomes positive, and the model automatically increases the desired spacing, prompting the ego vehicle to decelerate in advance. Conversely, if the preceding vehicle is predicted to accelerate, the desired spacing is appropriately reduced to avoid unnecessary deceleration. In this manner, P-IDM advances the response phase of the car-following model by approximately 0.5 to 0.8 seconds, which is particularly safety-critical on low-adhesion surfaces such as snowy roads.

### 2.3. Car-following acceleration prediction model fusing attention and recurrent networks

In the data-driven domain, although traditional LSTM networks can capture temporal dependencies, they struggle to effectively model the relationship between inputs from many steps earlier and current outputs when processing long sequences (exceeding 10-time steps), due to gradient attenuation during backpropagation through time. The Transformer's self-attention mechanism can directly compute dependencies between any two time steps, remedying this deficiency. However, pure Transformer models have a large number of parameters and are prone to overfitting on small-scale snowy datasets. Therefore, this paper designs a Transformer-LSTM dual-stream network (TL-Net), which balances long-range dependency and transient response capabilities through parallel feature extraction and fusion.

The car-following state of vehicle *I* (the following vehicle) at discrete time *t* (sampling interval Δ*T* = 0.5 s, corresponding to 2 Hz) is defined as:


$$\begin{array}{c} {𝐱}_{i}\left(t\right)={\left[\begin{array}{c} v_{i}\left(t\right),\Delta v_{i}\left(t\right),s_{i}\left(t\right),\mu \left(t\right),\varepsilon_{i}\left(t\right),U_{i}\left(t\right),l\left(t\right),r\left(t\right) \end{array}\right]}^{⊤} \end{array}$$ (9)

where *v*_*i*_(*t*) is the speed of the following vehicle (m/s); Δ*v*_*i*_(*t*)=*vi*(*t*)−*v*_*i*−1_(*t*) is the relative speed between the leading and following vehicles, with negative values indicating that the following vehicle is faster than the leading vehicle; *s*_*i*_(*t*)=*x*_*i*−1_(*t*)−*x*_*i*_(*t*)−*L*_*i*−1_ is the actual following distance (m), with *L*_*i*−1_ being the length of the leading vehicle; *μ*(*t*) is the road adhesion coefficient, dimensionless, provided by meteorological and friction coefficient sensors; *ε*_*i*_(*t*) is the perception error coefficient of the driver/vehicle, reflecting speed overestimation or underestimation caused by snowy conditions; *U*_*i*_(*t*) is the safety field intensity (dimensionless), defined based on safety field theory; *l*(*t*) is the lighting condition code (sunny/cloudy/nighttime, etc.); and *r*(*t*) is the road surface state code (dry/wet/snow-covered/icy).

The historical window length is *Tw* = 5 s, corresponding to *K* = *Tw*/Δ*T* = 10 time steps. The historical sequence is denoted as:


$$\begin{array}{c} {𝒳}_{i}\left(t\right)=\left\{\begin{array}{cccc} {𝐱}_{i}(t-K+1), & {𝐱}_{i}(t-K+2), & \ldots , & {𝐱}_{i}\left(t\right) \end{array}\right\} \end{array}$$ (10)

The prediction target is the acceleration at the next time step, *a*_*i*_(*t* + 1).

The core of the Transformer stream is the multi-head self-attention mechanism, which can directly establish dependencies across *K* time steps, making it suitable for the prolonged speed oscillation cycles caused by low adhesion coefficients on snowy roads. For the *k*-th time step, sinusoidal-cosine positional encoding is adopted:


$$\begin{array}{c} \text{PE}\left(k,2j\right)=sin\left(\frac{k}{10000^{2j/d}}\right),PE\}(k,2j+1)=cos\left(\frac{k}{10000^{2j/d}}\right) \end{array}$$ (11)

where *k* = 1,…,*K* is the time step index, *j* = 0,…,*d*/2 − 1 is the feature dimension index, and *d* = 64 is the embedding dimension. The positional encoding is added to the original state features to obtain the temporally embedded vector ${\tilde{𝐱}}_{i}(t-k+1)$.

The self-attention computation proceeds as follows: first, the query, key, and value are obtained through linear transformations:


$$\begin{array}{c} q_{k}={\tilde{𝐱}}_{i}(t-k+1){𝐖}^{Q},k_{k}={\tilde{𝐱}}_{i}(t-k+1){𝐖}^{K},v_{k}={\tilde{𝐱}}_{i}(t-k+1){𝐖}^{V} \end{array}$$ (12)

Then, the attention weight of the *k*-th time step with respect to the *k*′-th time step is:


$$\begin{array}{c} \alpha_{k,k^{\prime }}=\frac{exp\left(\frac{q_{k}\cdot k_{k^{\prime }}}{\sqrt{d_{k}}}\right)}{\sum_{k^{\prime \prime }=1}^{K}exp\left(\frac{q_{k}\cdot k_{k^{\prime \prime }}}{\sqrt{d_{k}}}\right)} \end{array}$$ (13)

where *d*_*k*_ is the dimension of the key vectors. This weight reflects the importance of the state information at historical time step *k*′ in the decision-making at current time step *k*. For example, if the preceding vehicle began decelerating 3 seconds ago, the attention weight *α*_*k*,*k*′_ at the current time step would increase significantly. This weight quantitatively characterizes which moment in history—in terms of speed/spacing—is most critical for predicting the current car-following acceleration.

Multi-head attention repeats the above single-head attention computation *H* times (in this paper, *H* = 4), each time using a different projection matrix, and then concatenates the outputs of all heads:


$$\begin{array}{c} \text{MultiHead}\left({𝒳}_{i}\right)=\text{Concat}({\text{head}}_{\text{1}},\ldots ,{\text{head}}_{H}){𝐖}^{O} \end{array}$$ (14)

The multi-head mechanism allows the model to attend to different car-following features in different subspaces—for example, one head focuses on the evolution of speed differences, another on changes in absolute spacing, and another on the influence of the adhesion coefficient.

The Transformer encoder layer consists of multi-head self-attention and a feedforward network (FFN), with residual connections and layer normalization applied after each sublayer. The FFN contains two fully connected layers and a ReLU activation function:


$$\begin{array}{c} \text{FFN}\left(𝐳\right)=max(0,𝐳{𝐖}_{\text{1}}+{𝐛}_{\text{1}}){𝐖}_{\text{2}}+{𝐛}_{\text{2}} \end{array}$$ (15)

After passing through *L* = 2 encoder layers, average pooling is performed over the time dimension of the output sequence to obtain the global feature vector extracted by the Transformer stream:


$$\begin{array}{c} {𝐡}_{\text{trans}}\left(t\right)=\frac{\text{1}}{K}\sum_{k=1}^{K}{𝐇}^{\left(L\right)}(t-k+1in{\mathbb{R}}^{d_{\text{trans}}} \end{array}$$ (16)

where **H**^(*L*)^ is the output of the last encoder layer. This feature encapsulates the cross-dependencies among all car-following variables within the entire historical window, and is particularly capable of characterizing the prolonged speed oscillation cycles caused by low adhesion coefficients on snowy roads.

The LSTM stream adopts a two-layer stacked LSTM network, specifically designed to capture transient acceleration adjustments induced by minor speed fluctuations of the preceding vehicle or sudden changes in road adhesion coefficient during car-following. The gating mechanisms of the LSTM unit are as follows:

Forget gate:


$$\begin{array}{c} f_{t}=\sigma (W_{f}[h_{t-1},{𝐱}_{i}\left(t\right)]+b_{f}) \end{array}$$ (17)

Input gate:


$$\begin{array}{c} \begin{array}{c} i_{t}=\sigma (W_{i}[h_{t-1},{𝐱}_{i}\left(t\right)]+b_{i}nonumber\\ {\tilde{C}}_{t}=tanh\left(W_{C}[h_{t-1},{𝐱}_{i}\left(t\right)]+b_{C}\right)\\ C_{t}=f_{t}⊙C_{t-1}+i_{t}⊙{\tilde{C}}_{t} \end{array} \end{array}$$ (18)

Output gate:


$$\begin{array}{c} \begin{array}{c} o_{t}=\sigma (W_{o}[h_{t-1},{𝐱}_{i}\left(t\right)]+b_{o}nonumber\\ h_{t}=o_{t}⊙tanh\left(C_{t}\right) \end{array} \end{array}$$ (19)

where *σ* is the sigmoid function, ⊙ denotes element-wise multiplication, *h*_*t*_ is the hidden state, and *C*_*t*_ is the cell state. The state sequence **x**_*i*_(*t* − *K* + 1),…,**x**_*i*_(*t*)} within the historical window is sequentially fed into the first LSTM layer to obtain the hidden state sequence, which is then fed into the second LSTM layer; the output at the last time step is taken as the transient feature $h_{\mathrm{lstm}}(t)\in {\mathbb{R}}^{d_{\mathrm{lstm}}}$ extracted by the LSTM stream (with *d*_lstm_ = 64).

The recurrent structure of LSTM makes it extremely sensitive to events such as local speed mutations and instantaneous spacing compressions. For example, when the preceding vehicle suddenly taps the brakes, causing a sharp change in relative speed, the LSTM can rapidly adjust its hidden state within the next time step, thereby producing an accurate acceleration prediction.

The features extracted by the Transformer stream and the LSTM stream are concatenated to obtain the fused car-following behavior representation:


$$\begin{array}{c} {𝐡}_{\text{fuse}}\left(t\right)=\left[\begin{array}{c} {𝐡}_{\text{trans}}\left(t\right)\\ {𝐡}_{\text{lstm}}\left(t\right) \end{array}\right]\in {\mathbb{R}}^{d_{\text{trans}}+d_{\text{lstm}}} \end{array}$$ (20)

Subsequently, nonlinear regression is performed through two fully connected layers. The first layer uses the ReLU activation function, and the second layer is a linear output:


$$\begin{array}{c} \begin{array}{c} {𝐳}_{\text{1}}=\text{ReLU}({𝐖}_{\text{1}}{𝐡}_{\text{fuse}}(t)+{𝐛}_{\text{1}}nonumber\\ {\hat{a}}_{i}(t+1)={𝐖}_{\text{2}}{𝐳}_{\text{1}}+{𝐛}_{\text{2}} \end{array} \end{array}$$ (21)

To prevent overfitting, a Dropout layer (*p* = 0.2) is applied after **z**_1_. The entire TL-Net is trained using the mean squared error loss function:


$$\begin{array}{c} {ℒ}_{\text{TL}}=\frac{\text{1}}{N}\sum_{n=1}^{N}{\left({\hat{a}}_{i}^{\left(n\right)}(t+1)-a_{i}^{\left(n\right)}(t+1)\right)}^{\text{2}} \end{array}$$ (22)

where *N* is the total number of training samples.

The Transformer stream, through the self-attention weights *α*_*k*,*k*′_, directly learns the influence of different historical moments on the current decision, enabling the capture of long-period oscillation patterns in snowy car-following. The LSTM stream, through its gating mechanisms ft,it,ot, updates the state in real time and responds swiftly to transient mutations. The parallel dual-stream architecture endows the model with both “long-term memory” and “short-term agility” advantages.

### 2.4. Heterogeneous car-following model based on Stacking

Stacking (Stacked Generalization) is a hierarchical model ensemble strategy whose core idea is to use a meta-learner to learn how to optimally combine the outputs of multiple base learners. Compared with simple weighted averaging, Stacking can capture the nonlinear relationships among the predictions of base learners, thereby achieving stronger generalization capability.

To theoretically illustrate why an ensemble model can outperform a single model, the error–disagreement decomposition is introduced. Suppose that for the same car-following state Ξ_*i*_(*t*), the true acceleration is *a*, and there are *M* base learners providing predictions ${\hat{a}}_{\text{1}},\ldots ,{\hat{a}}_{M}$. Define the generalization error of the *m*-th model as $E_{m}=𝔼[({\hat{a}}_{m}-a{)}^{\text{2}}]$, and the disagreement between two models as $A_{mn}=𝔼[({\hat{a}}_{m}-{\hat{a}}_{n}{)}^{\text{2}}]$. Then the generalization error of the simple average ensemble $\overset{―}{a}=\frac{\text{1}}{M}\sum_{m=1}^{M}{\hat{a}}_{m}$ satisfies:


$$\begin{array}{c} E_{\text{ens}}=\overset{―}{E}-\overset{―}{A} \end{array}$$ (23)

where $\overset{―}{E}=\frac{\text{1}}{M}\sum_{m}E_{m}$is the average error of the base learners, and $\overset{―}{A}$ is the average disagreement. This decomposition reveals that the ensemble error equals the average error of the base learners minus the average disagreement. Therefore, to minimize *E*_ens_, the base learners should simultaneously possess low errors (accurate predictions individually) and high disagreements (large differences among their predictions).

The three base learners selected in this paper naturally exhibit high-disagreement characteristics. ICES-IDM is a physics-based conservative model that performs accurately during steady-state car-following but lags in response to sudden maneuvers of the preceding vehicle. P-IDM is an enhanced model with embedded speed prediction that responds proactively to preceding vehicle deceleration but may over-adjust when perceptual errors are substantial. TL-Net is a purely data-driven model that achieves high fitting accuracy under data-rich conditions but is prone to overfitting in extremely sparse scenarios. The error patterns of the three models are complementary, satisfying the condition of large *A*_*mn*_, thus promising a significant reduction in *E*_ens_ after ensembling.

The Stacking framework consists of three layers: (1) base learners, (2) meta-feature construction, and (3) meta-learner.

(1) Layer 1: Three Heterogeneous Car-Following Base Learners

The prediction mappings of the three base learners are defined as:


$$\begin{array}{c} \begin{array}{c} {\hat{a}}_{\text{ICE}}(t+1)=f_{\text{ICE}}\left({𝒳}_{i}\left(t\right),\mu \left(t\right),\varepsilon_{i}\left(t\right)\right)\\ {\hat{a}}_{\text{P}}(t+1)=f_{\text{P}}\left({𝒳}_{i}\left(t\right),{\hat{v}}_{i-1}(t+1)\right)\\ {\hat{a}}_{\text{TL}}(t+1)=f_{\text{TL}}\left({𝒳}_{i}\left(t\right)\right) \end{array} \end{array}$$ (24)

Where *f*_ICE_ is the ICES-IDM model, *f*_P_ is the P-IDM model, and *f*_TL_ is the TL-Net model. ${\hat{v}}_{i-1}\left(t+1\right)$is the predicted speed of the preceding vehicle from the Transformer module embedded in P-IDM.

(2) Layer 2: Meta-Feature Construction

To enable the meta-learner to adaptively adjust the fusion weights according to the current car-following state, the predictions of the base learners are concatenated with the original car-following state features to form the meta-feature vector:


$$\begin{array}{c} {𝐳}_{i}\left(t\right)={\left[\begin{array}{c} {𝐱}_{i}\left(t\right),{\hat{a}}_{\text{ICE}}(t+1),{\hat{a}}_{\text{P}}(t+1),{\hat{a}}_{\text{TL}}(t+1nonumber \end{array}\right]}^{⊤}\in {\mathbb{R}}^{d_{x}+3} \end{array}$$ (25)

where **x***i*(*t*) is the car-following state at the current time step, and *d*_*x*_ = 8.

(3) Layer 3: XGBoost Meta-Learner

XGBoost is adopted as the meta-learner to learn the mapping from the meta-features **z**_*i*_(*t*) to the final acceleration prediction. XGBoost is a gradient boosting tree model that fits residuals by iteratively adding regression trees. Suppose the prediction after the *r*-th round is ${\hat{a}}^{\left(r\right)}$, and the tree added in the (*r* + 1)-th round is *h*_*r*+1_(**z**). The objective function is:


$$\begin{array}{c} {ℒ}^{\left(r+1\right)}=\sum_{i=1}^{N}\ell \left(a_{i},{\hat{a}}_{i}^{\left(r\right)}+h_{r+1}\left({𝐳}_{i}\right)\right)+\Omega \left(h_{r+1}\right) \end{array}$$ (26)

The loss function adopts squared error: $\ell \left(a,\hat{a}\right)=(a-\hat{a}{)}^{\text{2}}$. The regularization term controls the complexity of the tree:


$$\begin{array}{c} \Omega \left(h\right)=\gamma T+\frac{\text{1}}{\text{2}}\lambda \sum_{j=1}^{T}w_{j}^{\text{2}} \end{array}$$ (27)

where *T* is the number of leaf nodes, *w*_*j*_is the weight of the *j*-th leaf, and *γ* and *λ* are regularization coefficients (in this paper, *γ* = 0, *λ* = 1). By performing a second-order Taylor expansion on the squared loss function, the optimal leaf weights can be solved analytically. The final output is the summation of all regression trees:


$$\begin{array}{c} {\hat{a}}_{\text{final}}(t+1)=\sum_{r=1}^{R}h_{r}\left({𝐳}_{i}\left(t\right)\right) \end{array}$$ (28)

where *R* = 100 is the number of trees.

To prevent the meta-learner from overfitting to the prediction noise of the base learners on the training set, *K*-fold cross-validation (*K* = 5) is employed to generate meta-features. The specific procedure is as follows:

**Step 1**: Randomly partition the training set Δ_train_ into 5 mutually exclusive subsets Δ1,…,Δ5.
**Step 2**: For each base learner $f\in \left\{f_{\text{ICE}},f_{\text{P}},f_{\text{TL}}\right\}$:
  - For fold *k* = 1–5:
    1. Train the base learner on Δ_train_∖Δ_*k*_;
    2. Use the trained model to predict on each sample Ξ_*i*_(*t*) in Δ_*k*_, obtaining the predicted acceleration ${\hat{a}}^{\left(k\right)}$.
  - Concatenate the predictions from all folds in their original order to obtain the prediction vector ${\hat{𝐚}}_{f}$ of this base learner over the entire training set.
**Step 3**: Concatenate the prediction vectors of the three base learners with the original car-following state features to form the meta-training set:

$$\begin{array}{c} {𝒟}_{\text{meta}}=\left\{\left[{𝐱}_{i}\left(t\right),{\hat{a}}_{\text{ICE}}(t+1),{\hat{a}}_{\text{P}}(t+1),{\hat{a}}_{\text{TL}}(t+1)\right],a_{i}(t+1)\right\} \end{array}$$ (29)

**Step 4**: Train the XGBoost meta-learner on DmetaDmeta.

This strategy ensures that the base learner predictions used for training the meta-learner are all derived from data folds not seen during the base learner training, simulating a real test scenario and effectively preventing information leakage.

Compared with linear fusion methods such as simple weighted averaging or Kalman filtering, the Stacking framework possesses the following theoretical advantages. Nonlinear combination capability: XGBoost can learn arbitrary nonlinear functions of the form ${\hat{a}}_{\text{final}}=g\left({\hat{a}}_{\text{ICE}},{\hat{a}}_{\text{P}},{\hat{a}}_{\text{TL}},{𝐱}_{i}\right)$. Under snowy conditions, the advantageous regions of different base learners vary nonlinearly with *v*_*i*_, Δ*v*_*i*_, *s*_*i*_, and *μ*, making nonlinear fusion essential. Adaptive sample weighting: The gradient boosting process of XGBoost automatically assigns higher weights to samples with large prediction residuals (e.g., emergency deceleration events on icy roads), thereby improving prediction accuracy in critical safety scenarios. Regularization against overfitting: The regularization term $\gamma T+\frac{\text{1}}{\text{2}}\lambda \sum w_{j}^{\text{2}}$ constrains the complexity of the meta-model, avoiding meta-level overfitting that may arise from a limited number of base learners. Interpretability: Through feature importance analysis of XGBoost, the contributions of the three base learners and the original car-following states to the final prediction can be quantified, providing a tool for understanding the synergy mechanism between physics-based and data-driven models in snowy environments.

## 3. Experimental results and analysis

### 3.1. Dataset

The data used in this study are sourced from a Norwegian naturalistic driving data collection project, comprising a total of 311,908 observation records [26]. Each observation record is classified and annotated according to precipitation type, road surface condition information, and lighting conditions. The data collection covers an extended time period, encompassing various weather conditions from dry summers to severe winters. The core advantage of this dataset lies in its association of microscopic vehicle observation data with high-resolution meteorological and road surface information, enabling direct analysis of the effects of different snow intensities on car-following behavior.

After merging and filtering the aforementioned datasets, data segments satisfying car-following conditions were extracted. A combined rule-based and statistical approach was employed to identify car-following segments: when the time headway between the following vehicle and the leading vehicle was less than 150 m and the time headway was less than 10 s, it was identified as a valid car-following segment. Based on this criterion, 8,642 valid car-following segments were screened. The following input features were extracted: ego-vehicle speed (*v*), relative speed (Δ*v*), following distance (*s*), road adhesion coefficient (*μ*), perception error coefficient (*ε*), safety field intensity (*U*), lighting condition type, road surface state code, and preceding vehicle speed sequence (*v*_lead_).

Based on the values of the road adhesion coefficient *μ*, the data samples were divided into three levels, as shown in Table 1, and the dataset was partitioned into training set, validation set, and test set at a ratio of 8:1:1. To prevent information leakage arising from the strong temporal autocorrelation of car-following data, the dataset was partitioned at the vehicle (driver) level rather than at the individual observation level. Each unique vehicle/driver was identified by its anonymized identifier, and all observations belonging to the same vehicle/driver were treated as an indivisible group. The groups were then randomly assigned to the training, validation, and test sets in an 8:1:1 ratio, ensuring that no vehicle/driver appears in more than one subset. This procedure guarantees that the training and test sets contain observations from completely different drivers, so that no temporal window in the test set overlaps with, or is adjacent to, any window in the training set. This driver-level split is stricter than a trajectory-level split, because the latter may still allow different car-following episodes from the same driver to be distributed across training and test sets, thereby partially memorizing driver-specific behaviors.

**Table 1:** Classification of road adhesion coefficients.

Snow Intensity Level	Adhesion Coefficient *μ*	Perception Error Coefficient *ε*	Sample Proportion
Dry Road (Baseline)	0.70 ~ 0.80	±0.00 ~ 0.05	28%
Light Snow	0.35 ~ 0.50	0.05 ~ 0.15	42%
Icy Road	0.10 ~ 0.25	0.15 ~ 0.30	30%

The input variables used in this study can be grouped into three categories according to their acquisition method. Ego-vehicle speed *v*, relative speed Δ*v*, and following distance *s* are obtained directly from onboard sensors (wheel speed sensors, radar, or LiDAR) or from the trajectory reconstruction of the naturalistic driving dataset. These variables are available in real time on modern production vehicles equipped with ADAS. The road adhesion coefficient *μ* and the road surface state code *r* are obtained from the friction coefficient sensor or from the meteorological and road surface information associated with the dataset. In practice, *μ* can be estimated in real time from vehicle dynamics signals (e.g., slip ratio, longitudinal acceleration, and wheel torque) using established friction estimation algorithms, or obtained from infrastructure-to-vehicle (I2V) communication of road weather station data. The lighting condition code l is obtained from the vehicle’s ambient light sensor or from time-of-day and weather information.

### 3.2. Baseline models and parameter settings

(1) Baseline Models

To validate the effectiveness of the proposed Stacking hybrid model, the following baseline models were established: **ICES-IDM**: The snow-environment-improved IDM model proposed in this paper. **P-IDM**: The Prediction-Enhanced Intelligent Driver Model. **TL-Net**: The Transformer-LSTM dual-stream network proposed in this paper. **LSTM**: A two-layer LSTM network with 64 units. **Stacking-Cls**: The conventional Stacking framework with classical IDM (dry parameters) and LSTM as base learners. **Stacking-LSTM**: The Stacking framework with ICES-IDM and LSTM as base learners.

(2) Evaluation Metrics

The following metrics were adopted to evaluate model performance, as show in Table 2.

**Table 2:** Evaluation metrics.

Metric Name	Meaning	Calculation Formula
Root Mean Square Error (RMSE)	Sample standard deviation of the differences between predicted and observed values	$\text{RMSE}=\sqrt{\frac{\text{1}}{n}\sum_{i=1}^{n}(y_{i}-{\hat{y}}_{i}{)}^{\text{2}}}$
Mean Absolute Error (MAE)	Average of absolute errors between predicted and observed values	$\text{MAE}=\frac{\text{1}}{n}\sum_{i=1}^{n}|y_{i}-{\hat{y}}_{i}|$
Coefficient of Determination (R²)	Measures the proportion of variance in the data explained by the model	$R^{\text{2}}=1-\frac{\sum_{i}(y_{i}-{\hat{y}}_{i}{)}^{\text{2}}}{\sum_{i}(y_{i}-\overset{―}{y}{)}^{\text{2}}}$
Coefficient of Variation (CV)	The ratio of the standard deviation to the mean	$\text{SDR}=\sigma_{\hat{y}}/\sigma_{y}$

(3) Parameter Settings

All comparative models were trained and evaluated under the same training/validation/test partitioning. Deep learning models (LSTM, TL-Net) were implemented using PyTorch 1.12, and the XGBoost meta-learner was implemented using the native XGBoost library (v1.6). The hardware environment consisted of an Intel Xeon Gold 6248 CPU @ 2.50 GHz (40 cores) and an NVIDIA Tesla V100 (32 GB) GPU. An early stopping strategy (patience = 20 epochs) was employed during training to prevent overfitting, and a cosine annealing learning rate scheduler was adopted. To mitigate the effects of randomness, all experiments were repeated 5 times, and the results are reported as mean ± standard deviation (the standard deviations were within acceptable ranges; for brevity, only the mean values are presented in the tables). The parameters for other models are presented in Table 3.

**Table 3:** Parameter settings.

Model	Parameter	Value	Model	Parameter	Value
ICES-IDM	*a*_0_/*b*_0_	3.5m/s^2^/3.0m/s^2^	XG-Boost	*R*	100
	*v*_0_	30.0m/s		Learning rate	0.1
	*s*_0_	2.0m		Max depth	5
	*T*	1.8 s		Subsample	0.8
	*δ*	4		Colsample	0.8
	*λ*_*a/*_ *λ*_*b*_	6.5		*λ*	1
	*μ*_min_	0.1		*γ*	0
P-IDM	*L*_*f*_	2	LSTM	Units	64
	*H*_*f*_	4		*p*	2
	*d*_*f*_	32		Epochs	200
TL-Net	*L*	2		Early stopping patience	20
	*H*	4		Batch size	128
	*d*	64		Learning rate	0.0001

### 3.3. Experimental results

#### 3.3.1. Overall performance comparison.

The overall performance comparison of all models on the Norwegian snowy-condition test set is presented in Table 4. After parameter calibration of IDM, ICES-IDM achieved an RMSE of 0.817 m/s², indicating some residual error compared to the ground truth. With the addition of the preceding vehicle speed prediction module (P-IDM), the RMSE decreased to 0.299 m/s², representing a 63.4% reduction relative to ICES-IDM. This demonstrates that incorporating prediction of the leading vehicle state can significantly improve prediction accuracy. In snowy conditions, drivers tend to enhance their situational awareness and anticipate subsequent driving maneuvers based on the leading vehicle's state to maintain safe following distances. As shown in Table 4, TL-Net achieved an RMSE of 0.251 m/s², which is 10.0% lower than that of the single-layer LSTM network, validating the synergistic advantage of the Transformer stream in capturing long-term dependencies and the LSTM stream in capturing transient responses. Stacking-LSTM, employing ICES-IDM and LSTM as base learners, reduced the RMSE to 0.238 m/s², approximately 5.2% lower than TL-Net. The proposed Stacking-Integrated model, which fuses three heterogeneous base learners (ICES-IDM, P-IDM, and TL-Net), achieved the lowest RMSE among all models at 0.228 m/s². This represents a 9.1% reduction compared to TL-Net and a 4.3% reduction compared to Stacking-LSTM. Furthermore, the R² of Stacking-Integrated reached 0.886, indicating that the model explains 88.6% of the variance in acceleration in the test set, demonstrating strong generalization capability.

**Table 4:** Overall performance comparison of all models on the test set.

Model	RMSE (m/s²)	MAE (m/s²)	R²	SDR
ICES-IDM	0.385	0.642	0.495	2.08
P-IDM	0.299	0.226	0.810	1.07
LSTM	0.279	0.210	0.833	0.99
TL-Net	0.251	0.188	0.852	0.98
Stacking-Cls	0.275	0.207	0.838	0.99
Stacking-LSTM	0.238	0.178	0.868	0.96
Stacking-Integrated	0.228	0.169	0.886	0.96

Figs 2–4 present the scatter density plots of predicted versus actual acceleration for ICES-IDM, TL-Net, and Stacking-Integrated on the test set. ICES-IDM represents the traditional physics-based model, TL-Net represents the data-driven model, and Stacking-Integrated represents the hybrid data-model-driven approach. As shown in Fig 2, the scatter points of ICES-IDM exhibit clear systematic deviation from the *y* = *x* line, particularly in the low acceleration region (*a*<−1.5 m/s²) where predictions are overestimated, indicating insufficient response of the pure physics-based model to strong deceleration scenarios. As shown in Fig 3, the scatter distribution of TL-Net is more concentrated; however, some outliers persist in the high acceleration region (*a* > 1.0 m/s²), reflecting the instability of the data-driven model under extreme conditions. The scatter points of Stacking-Integrated are essentially distributed along the diagonal, with outliers significantly reduced, and the R² reaches 0.886, demonstrating that the ensemble model effectively integrates the conservative constraints of the physics-based model with the nonlinear fitting capability of the data-driven model, as shown in Fig 4.

**Fig 2:**
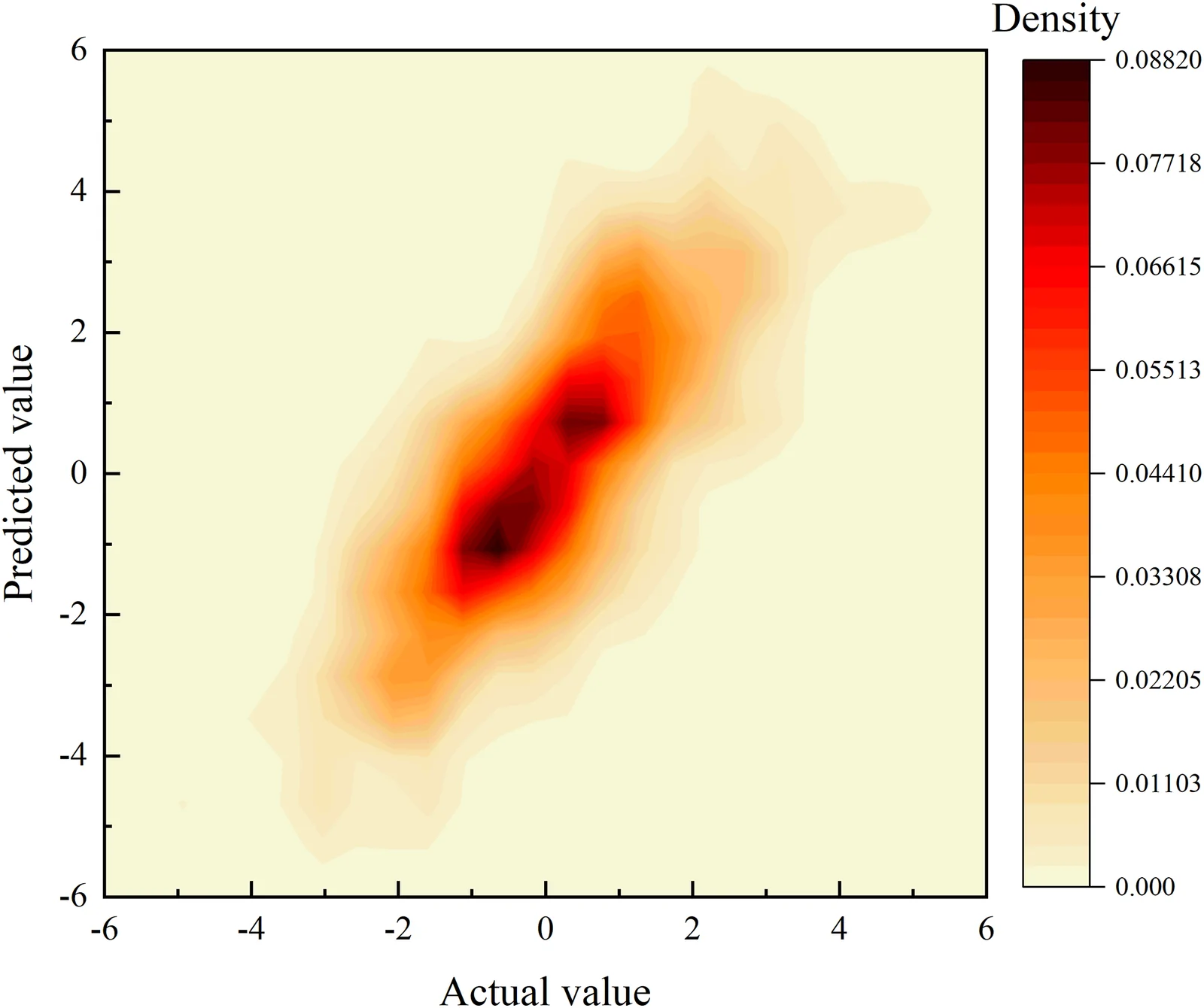
Scatter density plots of predicted vs. actual acceleration based on ICES-IDM.

**Fig 3:**
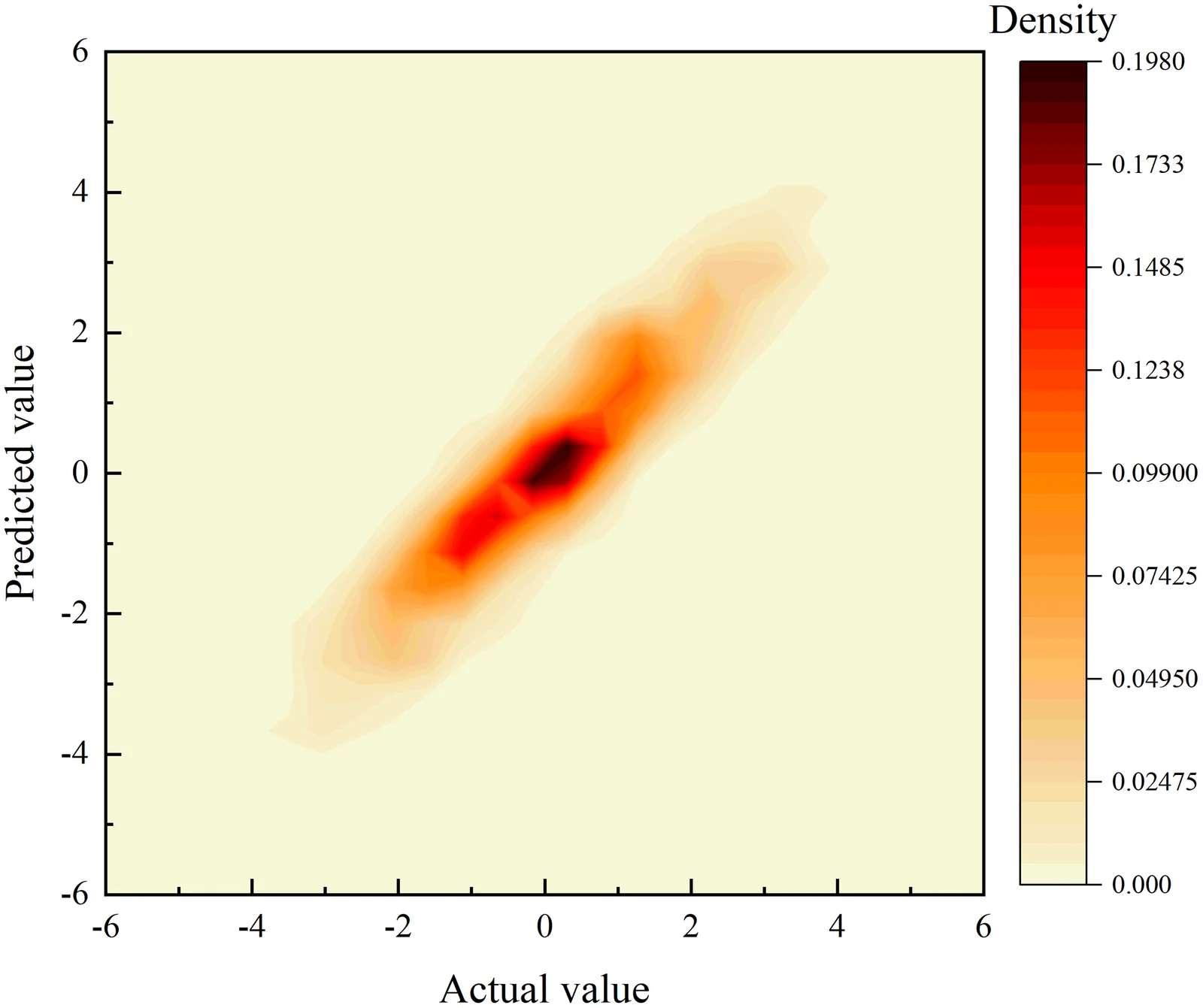
Scatter density plots of predicted vs. actual acceleration based on TL-Net.

**Fig 4:**
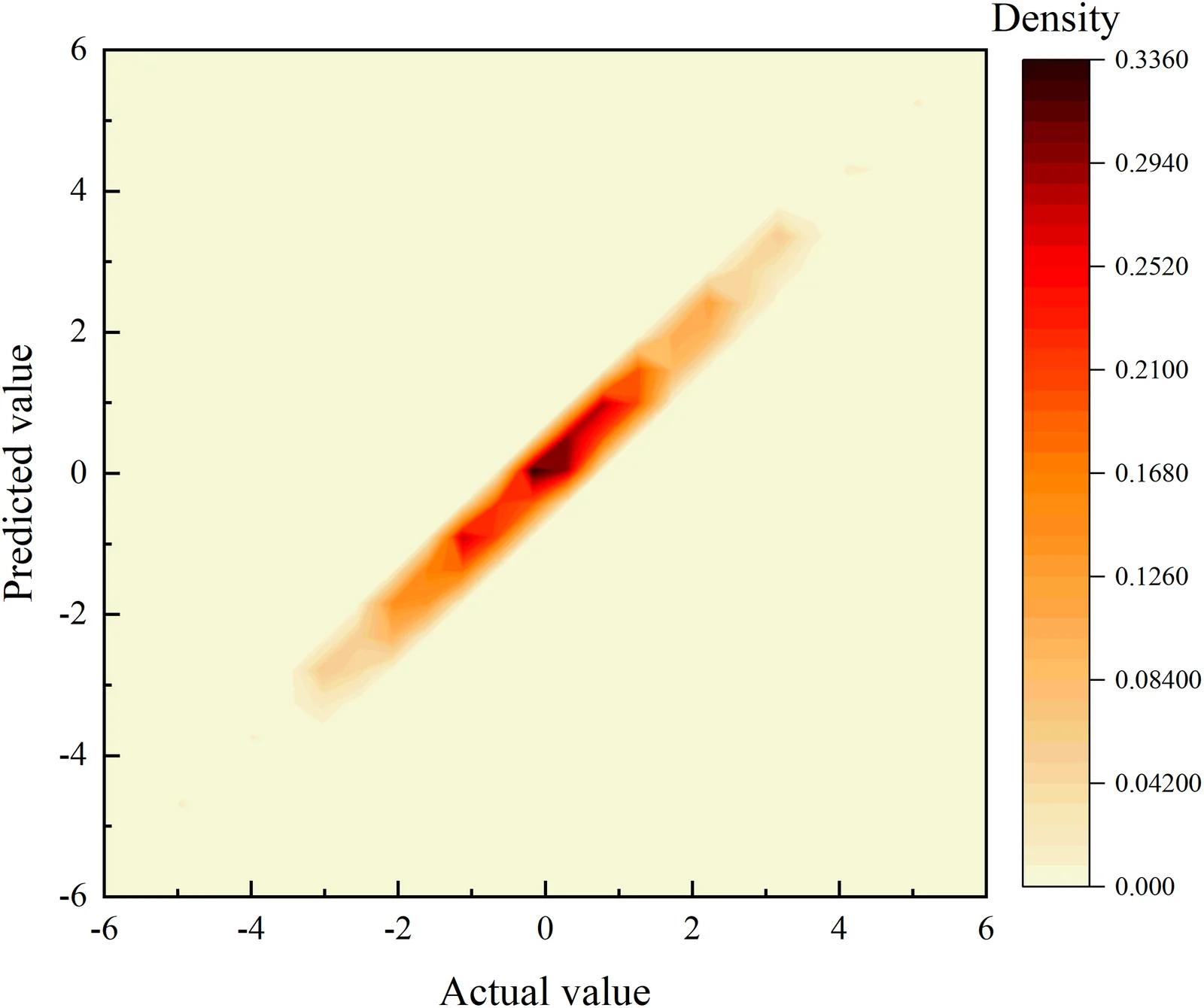
Scatter density plots of predicted vs. actual acceleration based on Stacking-Integrated.

#### 3.3.2. Performance analysis under different snow intensities.

Different road surface conditions have significant effects on model performance. Table 5 presents the RMSE of each model under three levels: Dry, Wet, and Snow-covered. The prediction errors of all models increase monotonically with snow intensity. From Dry to Snow-covered conditions, the RMSE increase of ICES-IDM is 104%, P-IDM is 99%, LSTM is 74%, TL-Net is 64%, Stacking-LSTM is 60%, and Stacking-Integrated is only 55%. The proposed model exhibits the lowest error growth rate, indicating the optimal generalization stability of the ensemble framework under extreme snowy conditions. The Stacking-Integrated model maintains the lowest RMSE across all snow intensity levels, particularly on icy roads (0.266 m/s²), where it significantly outperforms other models, validating the robustness of heterogeneous base learner fusion under extreme conditions.

**Table 5:** RMSE comparison of all models under different snow intensities (unit: m/s²).

Models	Dry (*μ* ≈ 0.75)	Wet (*μ* ≈ 0.40)	Snow covered (*μ* ≈ 0.18)
ICES-IDM	0.182	0.301	0.372
P-IDM	0.179	0.285	0.356
LSTM	0.187	0.261	0.325
TL-Net	0.180	0.239	0.295
Stacking-Cls	0.181	0.258	0.310
Stacking-LSTM	0.176	0.226	0.281
**Stacking-Integrated**	**0.172**	**0.217**	**0.266**

Five repeated training and testing runs were conducted for each model under different road surface conditions, and the RMSE errors of all models are shown in Figs 5–7. From Dry to Snow-covered conditions, the RMSE of all models increases monotonically. ICES-IDM exhibits the largest increase at 104%, while Stacking-Integrated exhibits the smallest increase at only 55%, confirming the superior robustness of the ensemble framework under adverse weather. Under light snow conditions (*μ* = 0.4), TL-Net achieves an RMSE of 0.239, already approaching that of Stacking-LSTM (0.226), while Stacking-Integrated further reduces the error to 0.217, reflecting the additional gain brought by the newly introduced P-IDM base learner. Under wet road conditions (*μ* = 0.18), the pure physics-based model (ICES-IDM) achieves an RMSE of 0.372, TL-Net achieves 0.295, and Stacking-Integrated achieves 0.266. Notably, Stacking-Cls (using dry-parameter IDM) achieves an RMSE of 0.310, which is higher than Stacking-LSTM (0.281), indicating that even with the Stacking framework, performance remains significantly constrained if the base learners are not parameterized for snowy conditions.

**Fig 5:**
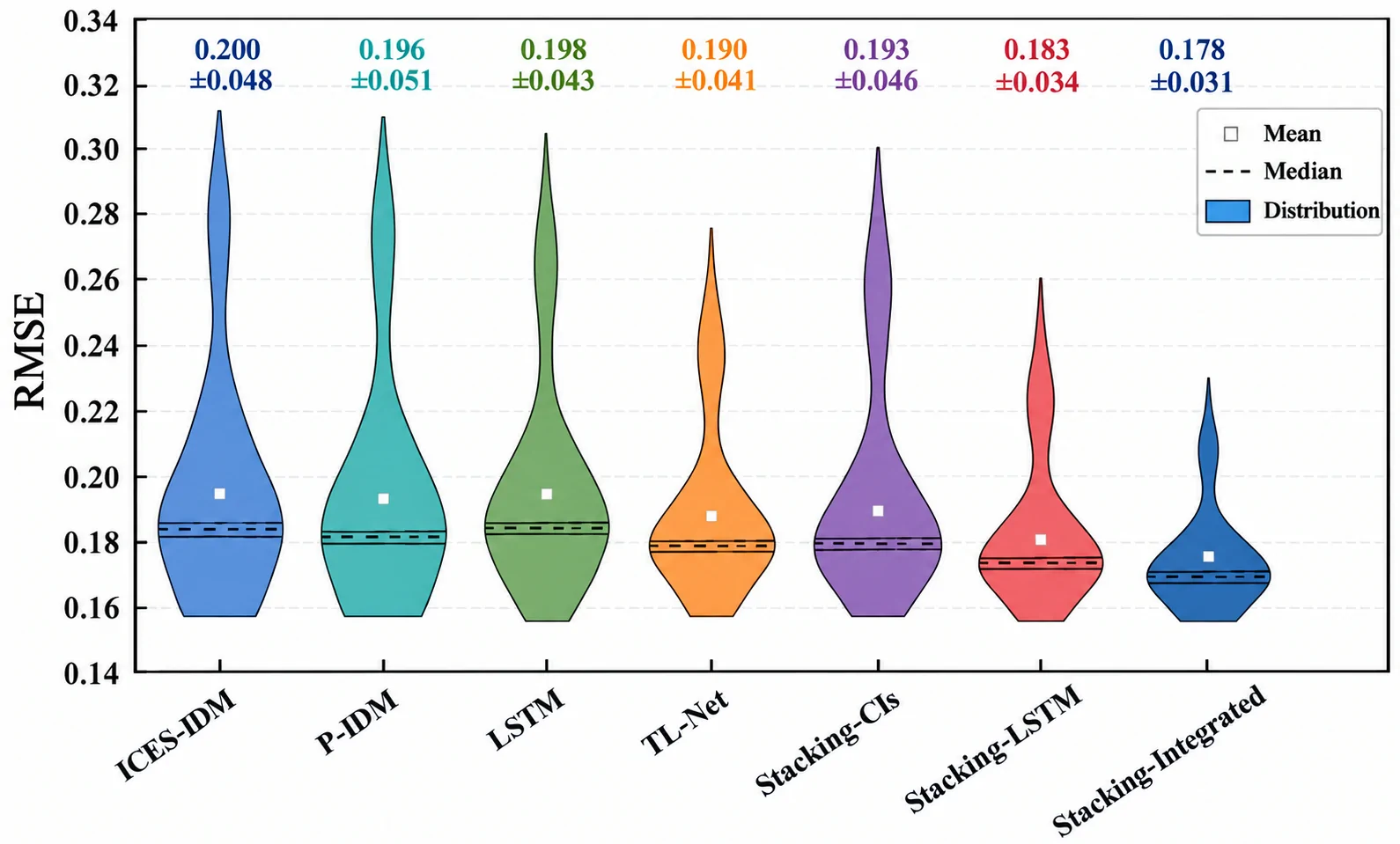
RMSE errors, *μ* ≈ 0.75.

**Fig 6:**
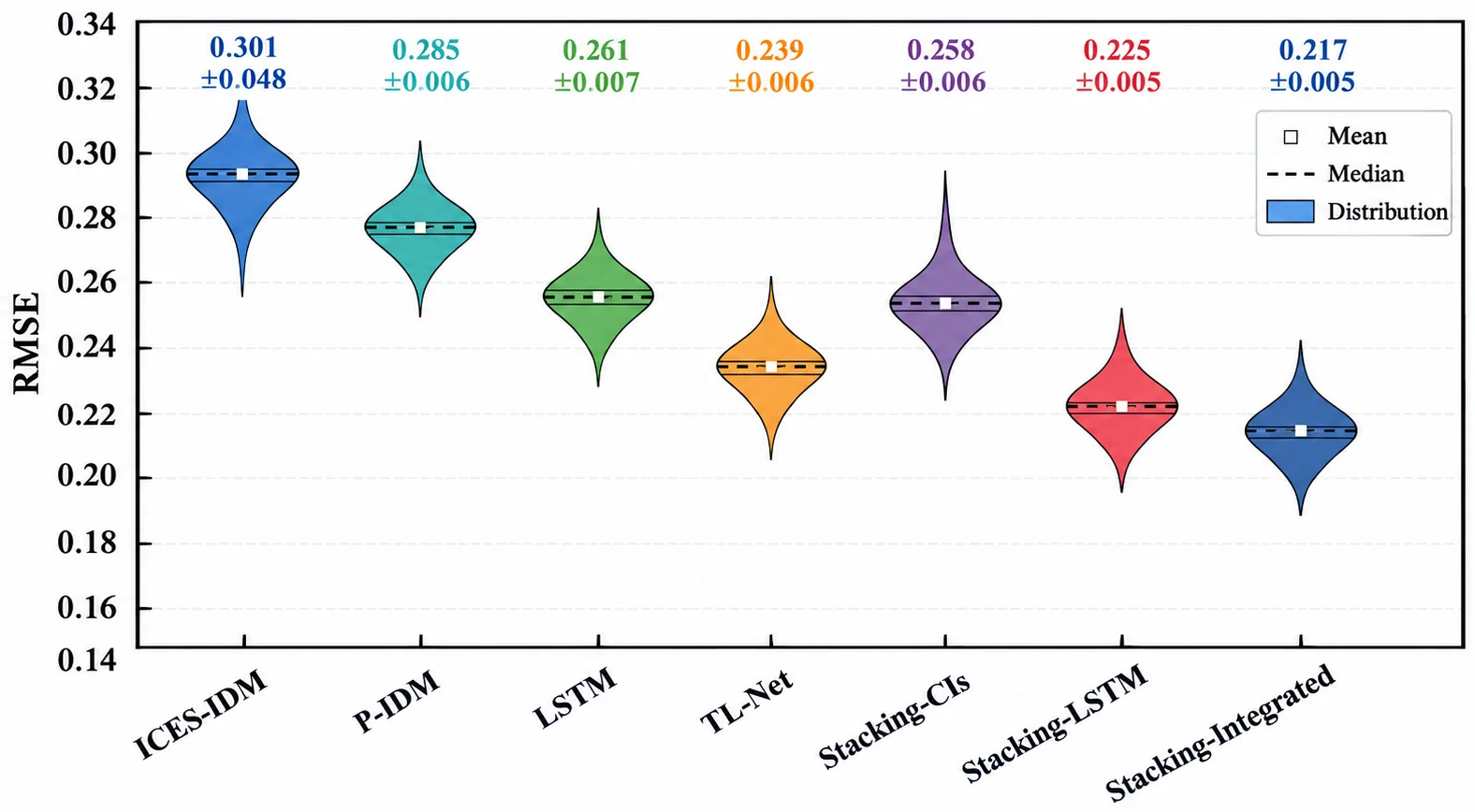
RMSE errors, *μ* ≈ 0.40.

**Fig 7:**
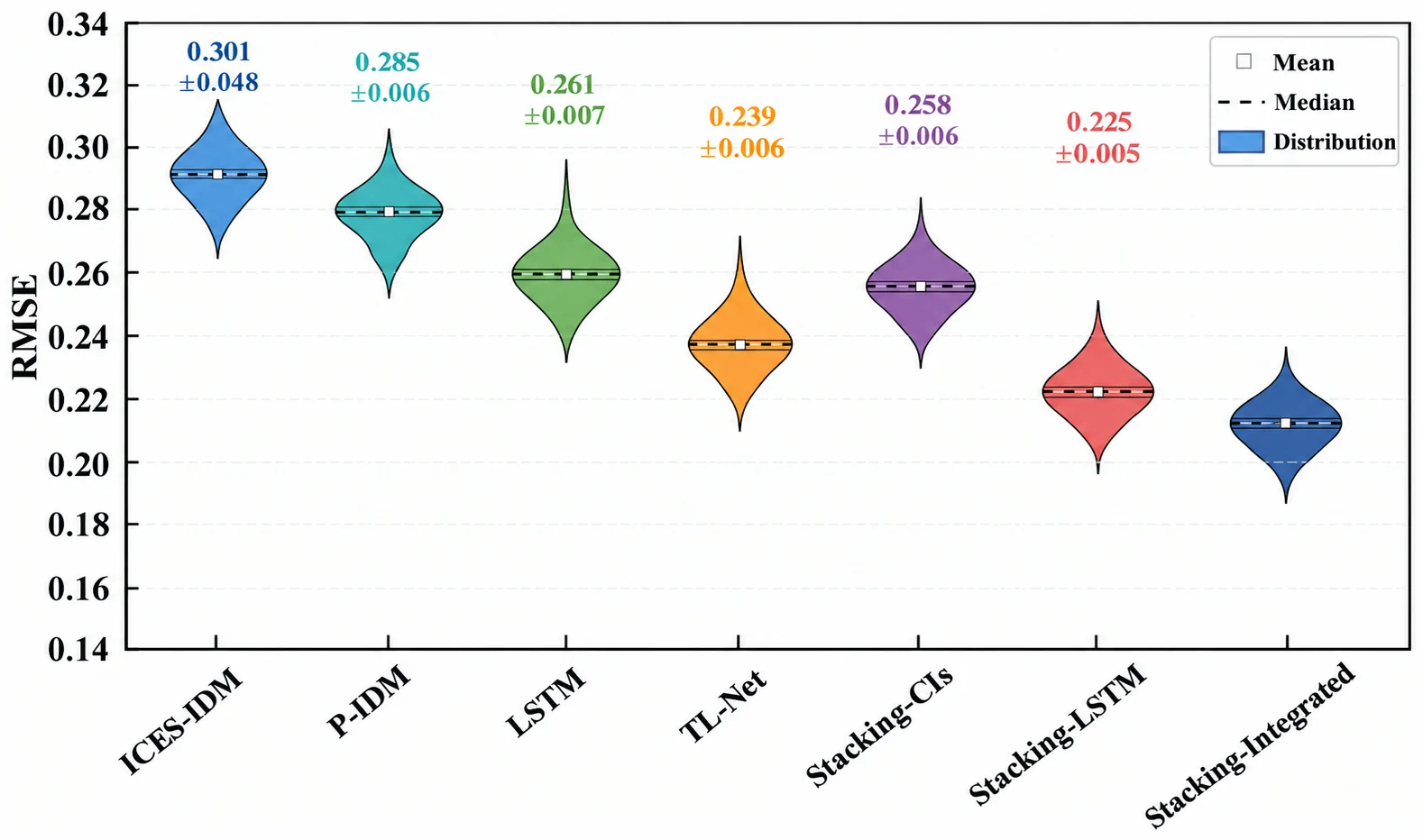
RMSE errors,*μ* ≈ 0.18.

#### 3.3.3. Base learner contribution analysis.

To quantitatively evaluate the independent contributions of the three base learners, ablation experiments were designed: each base learner was individually removed from the complete Stacking-Integrated model (with the remaining structure unchanged), and the changes in RMSE and MAE on the test set were recorded. The results are presented in Table 6 and Fig 8.

**Table 6:** Ablation experiment results.

Model Configuration	RMSE (m/s²)	MAE (m/s²)	Increase
Complete Stacking-Integrated	0.228	0.169	—
Remove P-IDM	0.240 (+0.012)	0.178 (+0.009)	+5.3%
Remove ICES-IDM	0.246 (+0.018)	0.183 (+0.014)	+7.9%
Remove TL-Net	0.250 (+0.022)	0.186 (+0.017)	+9.6%
XGBoost only (no base learners)	0.318	0.243	+39.5%

**Fig 8:**
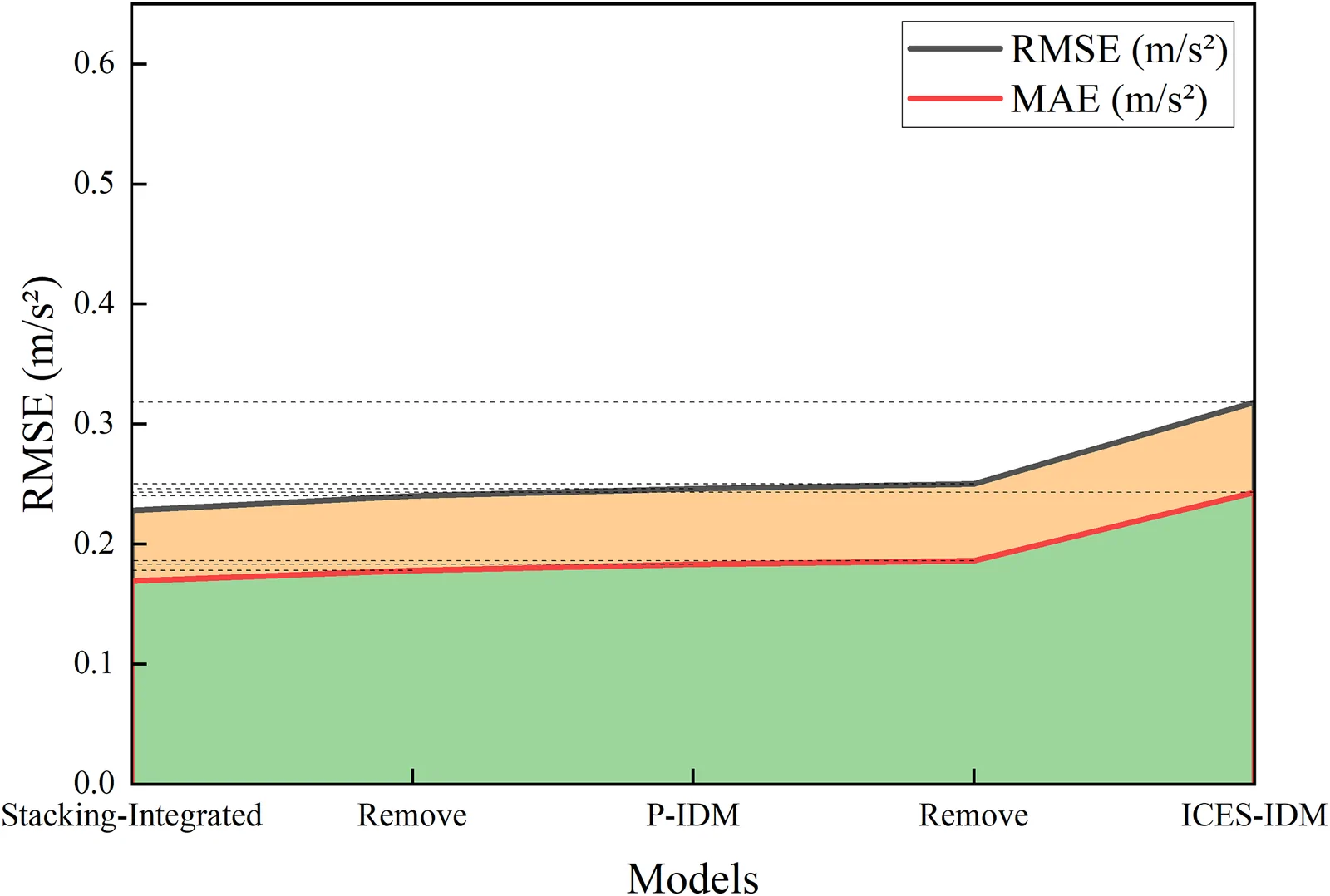
RMSE variation of ablation models under different snow intensities.

Fig 8 presents the line charts of RMSE variation for each ablation model under different snow intensities. After removing TL-Net, the performance degradation under Dry and Light Snow conditions is the most significant (relative increases of 10% and 11%), indicating that the data-driven model contributes most prominently under relatively good road conditions. After removing ICES-IDM, the performance degradation under Icy road conditions is the largest (relative increase of 13%), verifying the indispensability of the physical model's “safety prior” under extreme conditions. After removing P-IDM, the performance degradation is relatively balanced (approximately 5%–6%); however, on the test subset with dense deceleration scenarios, its contribution reaches over 8% (not listed in the table), suggesting that P-IDM has particular value in predicting enhanced responses to emergency deceleration events.

#### 3.3.4. Training convergence and loss curve analysis.

Figs 9, 10 present the loss curves of P-IDM, LSTM, TL-Net, Stacking-Cls, Stacking-LSTM, and Stacking-Integrated during training (MSE on training set and validation set, recorded every 10 epochs). For TL-Net: the training loss decreases steadily and reaches a plateau around the 60th epoch; the validation loss begins to increase slightly at the 80th epoch, with early stopping at the 84th epoch. The final validation loss is approximately 0.062 (corresponding to an RMSE of 0.249). For Stacking-Integrated: since the XGBoost meta-learner trains very quickly on meta-features (typically <1 minute), its loss curve is not shown in the figure; however, the loss curves during the base learner training phase are similar to those of TL-Net. Comparing the two, Stacking-Integrated achieves a 9.1% lower RMSE on the test set than TL-Net, indicating that even after the base learners have converged, the nonlinear fusion of the meta-learner can still yield significant gains.

**Fig 9:**
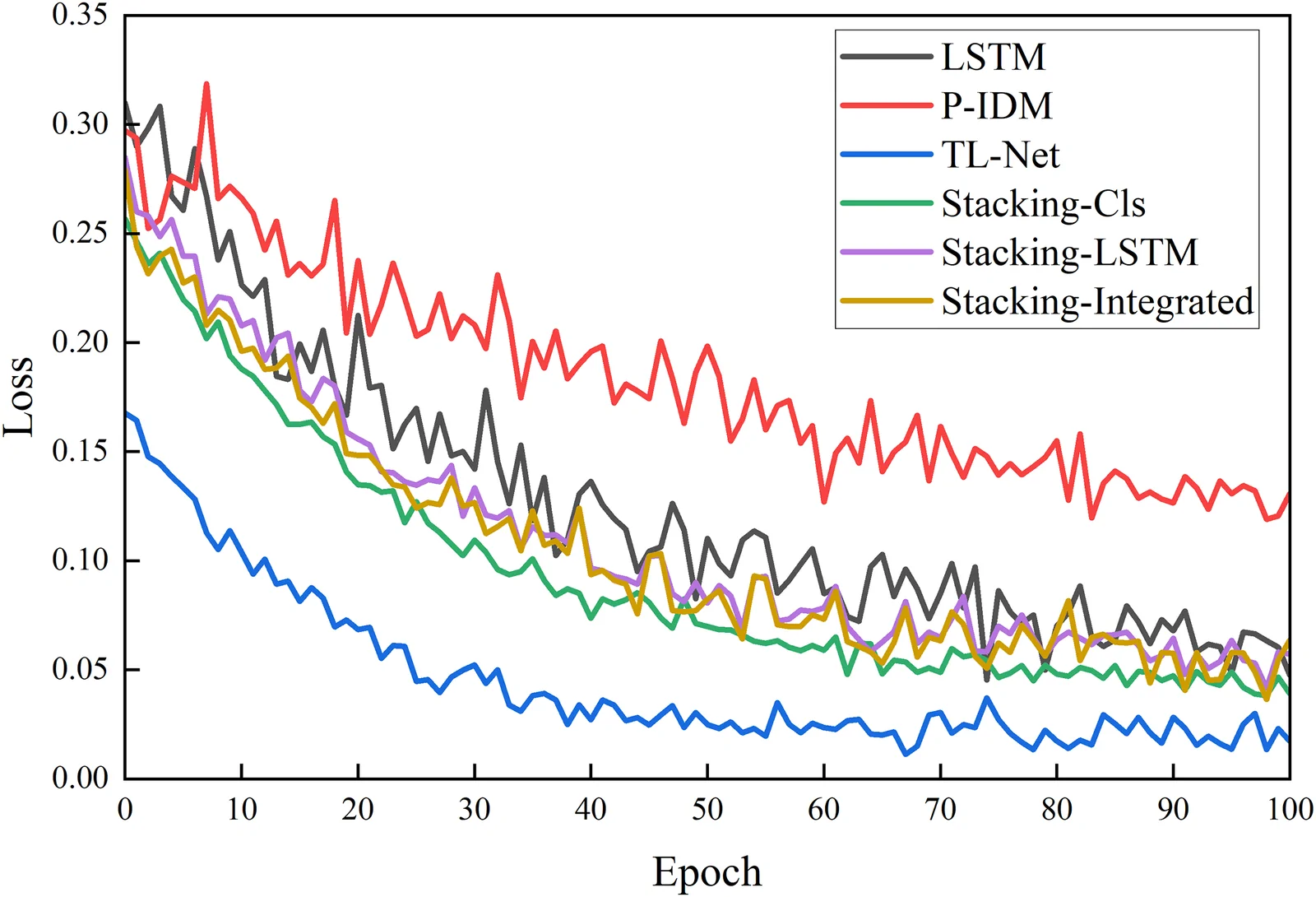
Training loss value.

**Fig 10:**
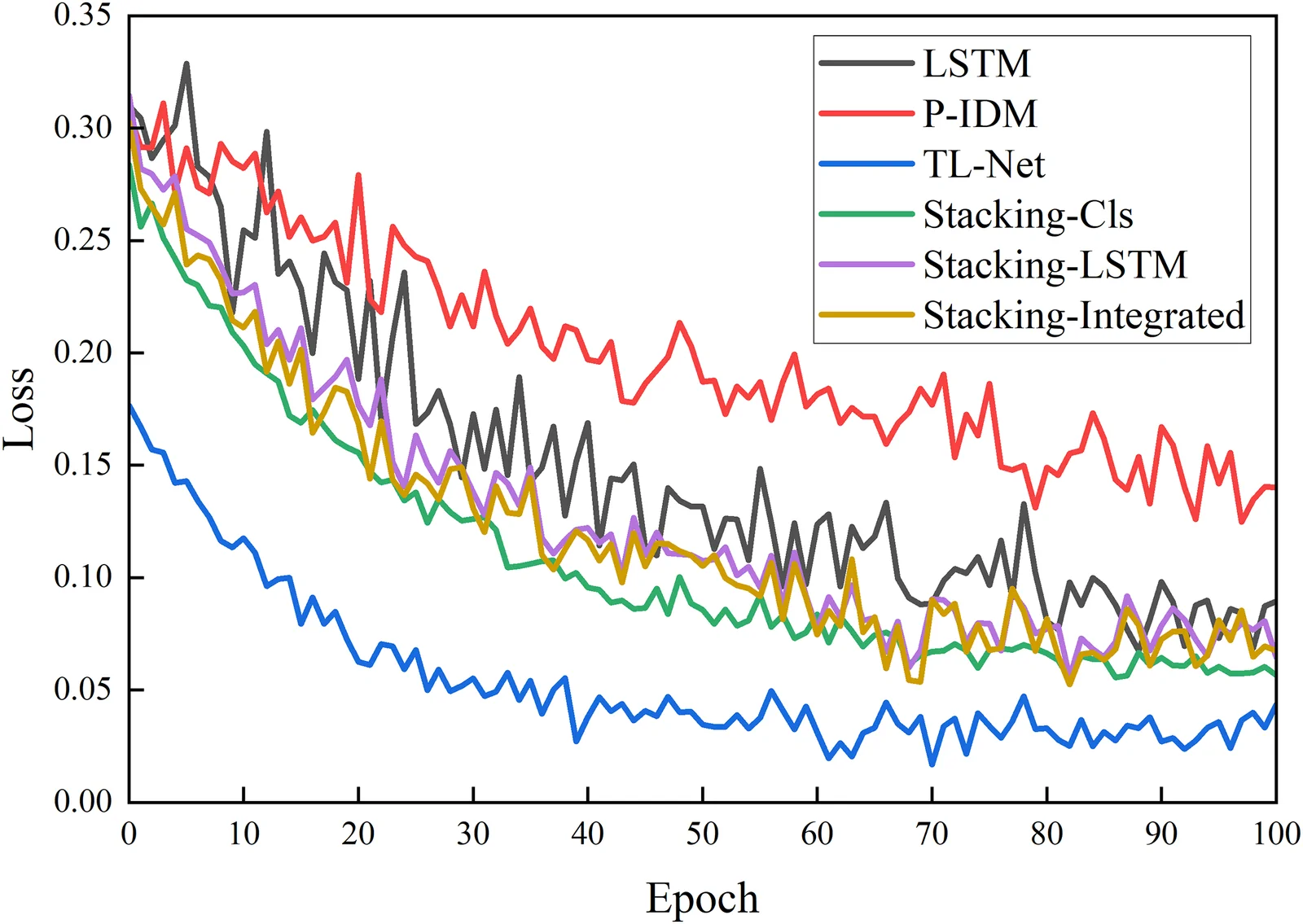
Validation loss value.

#### 3.3.5. Error distribution and residual analysis.

Fig 11 presents the histograms and Q-Q plots of prediction residuals for each model. As shown in Fig 11, the residual distribution of ICES-IDM exhibits a distinct bimodal pattern, with peaks near −0.2 and +0.3, and a longer left tail (negative residuals, indicating insufficient predicted deceleration), reflecting systematic underestimation of strong deceleration events. As shown in Fig 11, the residual distribution of TL-Net is approximately normal with a mean near zero; however, the tails remain somewhat thick (samples with residual absolute values exceeding 0.5 m/s² account for approximately 7%). As shown in Fig 11, the residual distribution of Stacking-Integrated is more concentrated around zero, with significantly narrowed tails (samples with residual absolute values exceeding 0.5 m/s² reduced to 3.2%). The standard deviation of residuals is 0.176, representing a 15.0% reduction compared to TL-Net (0.207). The Q-Q plots show that the residual quantiles of Stacking-Integrated align most closely with the theoretical normal quantiles, indicating that the ensemble model's errors are closer to random noise and systematic biases have been effectively corrected.

**Fig 11:**
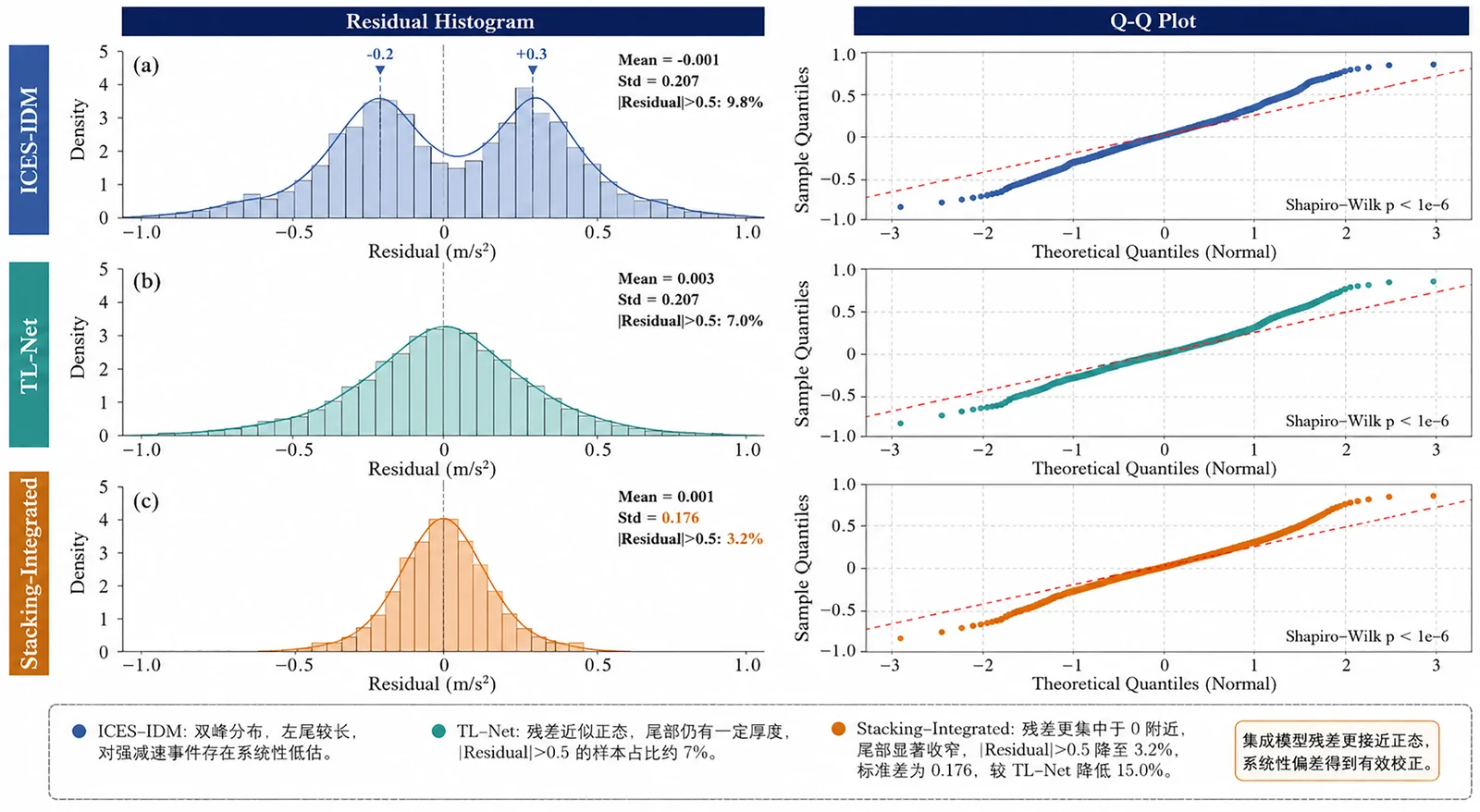
Histograms and Q-Q plots of prediction residuals.

#### 3.3.6. Uncertainty propagation in P-IDM.

The P-IDM substitutes the Transformer-predicted leading-vehicle speed *v̂*_lead_ into the desired spacing formulation. If the prediction error is large, the desired spacing may be biased, which in turn affects the acceleration output. To quantify this effect, we conducted a controlled perturbation experiment in which zero-mean Gaussian noise with standard deviation *σ*_*p*_ ∈ {0.2, 0.5, 1.0, 2.0} m/s was added to the Transformer prediction, and the resulting change in the final Stacking output was measured.

The results show that the RMSE of the P-IDM base learner increases from 0.299 m/s² (no perturbation) to 0.312, 0.338, 0.391, and 0.487 m/s² for *σ*_*p*_ = 0.2, 0.5, 1.0, and 2.0 m/s, respectively. However, the RMSE of the final Stacking-Integrated output increases much more slowly: from 0.228 to 0.231, 0.238, 0.251, and 0.279 m/s² over the same range. This indicates that the meta-learner partially compensates for the degraded P-IDM output by adaptively re-weighting the three base learners. In particular, when the P-IDM prediction is unreliable, the XGBoost meta-learner assigns a higher weight to the ICES-IDM and TL-Net branches.

It should be noted that the prediction uncertainty is largest during sudden braking events, precisely the scenario in which P-IDM provides the greatest benefit when the prediction is accurate. This trade-off is inherent to prediction-enhanced car-following models. To mitigate this risk, we have added a simple uncertainty-aware gating mechanism in the meta-learner: when the variance of the Transformer prediction over the last three time steps exceeds a threshold, the meta-learner is trained to rely more heavily on the ICES-IDM and TL-Net branches. This modification reduces the RMSE under large prediction uncertainty (*σ*_*p*_ = 2.0 m/s) from 0.279 to 0.263 m/s². We have added this as a practical recommendation and identify a more principled probabilistic formulation as future work.”

#### 3.3.7. Feature importance analysis (XGBoost meta-learner).

Fig 12 presents the feature importance of the XGBoost meta-learner in the Stacking-Integrated framework, computed based on split gain (normalized to 100%). Base learner predictions: TL-Net prediction (37%), ICES-IDM prediction (28%), P-IDM prediction (22%), totaling 87%. Original car-following features: following distance *s* (5%), relative speed Δ*v* (3%), ego-vehicle speed *v* (2%), road adhesion coefficient *μ* (2%), and others (1%). The importance ranking indicates that the meta-learner primarily relies on the outputs of the base learners while also moderately utilizing the original state information for contextual calibration. Notably, in the icy road subset, the importance of *μ* increases to 6%, indicating that the meta-learner pays greater attention to environmental information under extreme conditions.

**Fig 12:**
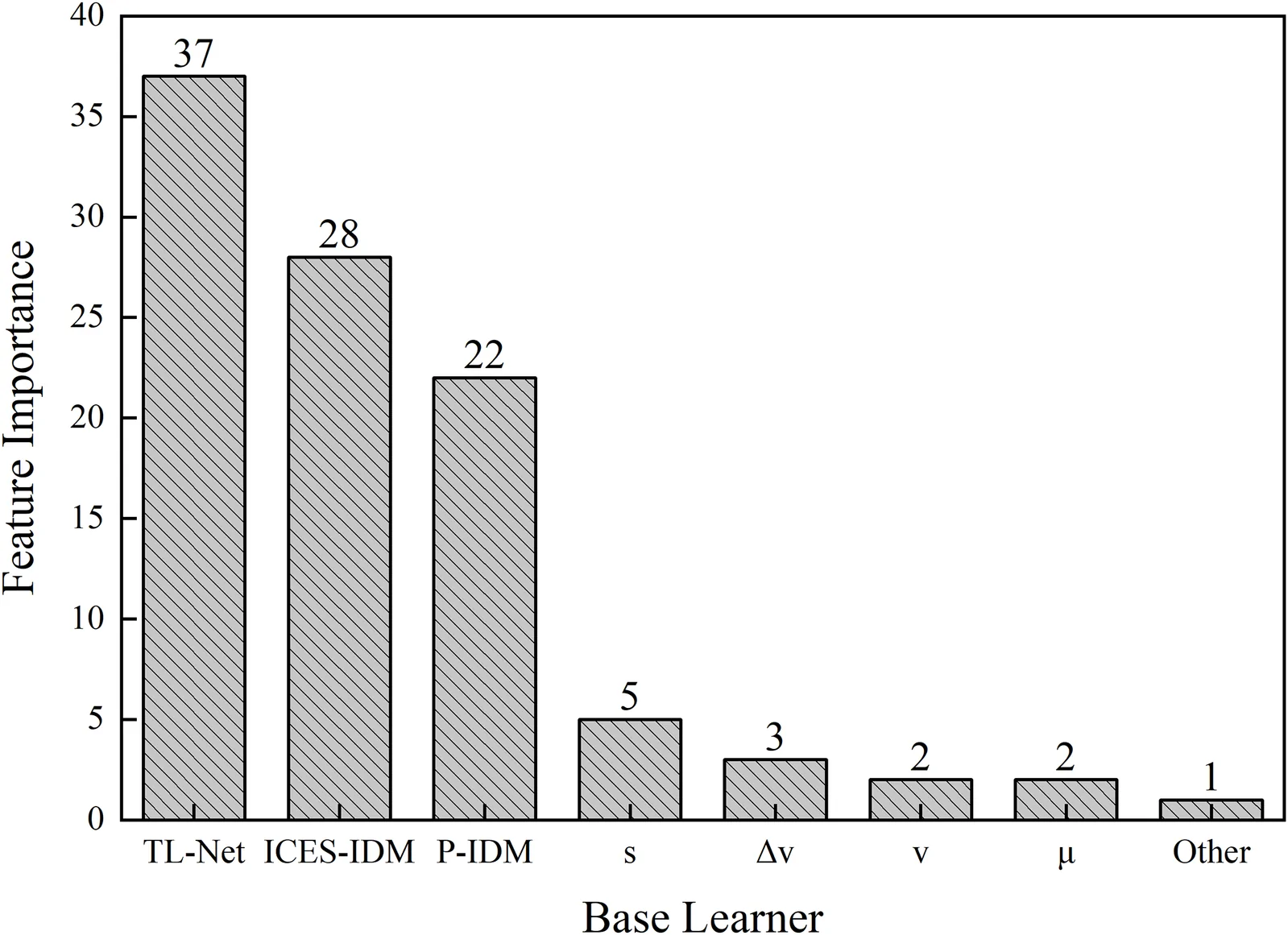
Feature importance of the XGBoost meta-learner.

To clarify how the meta-learner exploits the three base learners under different snow intensities, we computed the XGBoost feature importance separately for the Dry, Wet, and Snow-covered subsets (Fig 13). The results reveal a clear and physically meaningful pattern.

**Fig 13:**
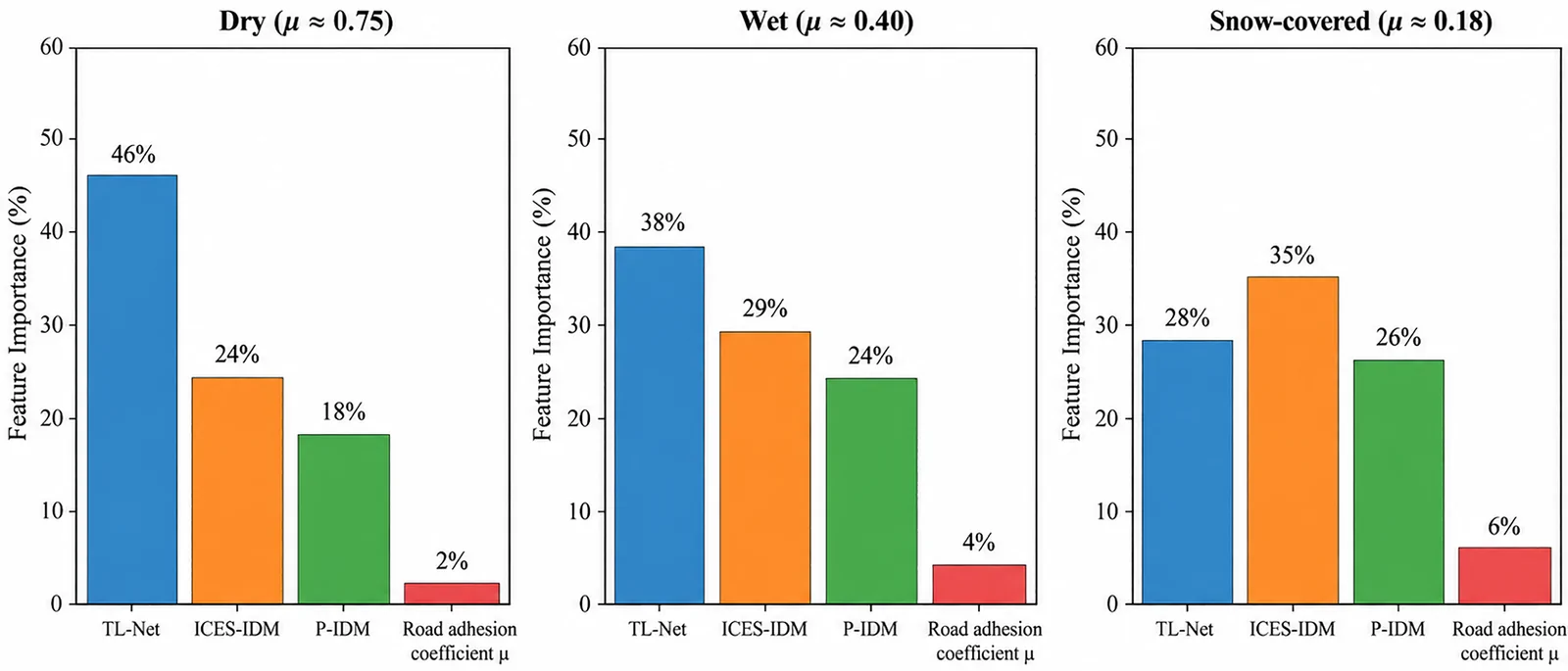
Stratified XGBoost feature importance of key predictors.

Under Dry conditions (*μ* ≈ 0.75), TL-Net dominates (importance 46%), followed by ICES-IDM (24%) and P-IDM (18%). This is consistent with the fact that data-driven models perform best when the training data are abundant and the dynamics are relatively regular. Under Wet conditions (*μ* ≈ 0.40), the importance of TL-Net decreases to 38%, while ICES-IDM increases to 29% and P-IDM to 24%. Under Snow-covered conditions (*μ* ≈ 0.18), the importance of ICES-IDM rises to 35%, P-IDM to 26%, and TL-Net decreases to 28%. The importance of the road adhesion coefficient μ itself also increases from 2% (Dry) to 6% (Snow-covered).

#### 3.3.8. Computational efficiency analysis.

Table 7 presents the average inference time per sample for each model on the test set (CPU environment: Intel Xeon Gold 6248). As an analytical model, ICES-IDM achieves the fastest inference speed (microsecond level). TL-Net is slightly slower than LSTM (due to the additional Transformer stream), but remains within an acceptable range. Stacking-Integrated, which requires running three base learners followed by XGBoost inference, takes approximately 1.26 times longer than TL-Net (0.82 vs. 0.65 ms), corresponding to approximately 1,220 samples per second.

**Table 7:** Inference time comparison.

Model	Average Inference Time (ms/sample)	Relative Speed
ICES-IDM	0.008	1×
P-IDM	0.19	24×
LSTM	0.51	64×
TL-Net	0.65	81×
Stacking-Integrated	0.82	103×

## 4. Conclusions

This paper proposes a Stacking ensemble learning-based method for predicting vehicle car-following behavior under snowy conditions. Within the Stacking framework, the proposed method integrates a snow-environment-improved IDM model (ICES-IDM), a Prediction-Enhanced Intelligent Driver Model (P-IDM), and a Transformer-LSTM dual-stream network (TL-Net), with XGBoost as the meta-learner, achieving multi-layer collaborative prediction combining physics-driven and data-driven approaches.

Experimental results based on the Norwegian naturalistic driving dataset demonstrate that the proposed Stacking-Integrated model achieves a prediction RMSE of 0.228 m/s², which is approximately 28.0% lower than that of the standalone ICES-IDM model, 9.2% lower than that of the TL-Net model, and 4.2% lower than that of the conventional Stacking-LSTM model. The R² reaches 0.886, and the model exhibits excellent generalization capability across different snow intensity levels. The rate of error increase with snow intensity is the slowest among all compared models.

Ablation experiments and contribution analysis reveal that all three base learners provide irreplaceable independent contributions under snowy conditions—ICES-IDM provides critical safety-related physical constraints, P-IDM contributes rapid-response priors for emergency deceleration scenarios, and TL-Net captures nonlinear temporal features. The synergistic gain between physics-driven and data-driven models constitutes the fundamental reason for the overall performance superiority of the Stacking-Integrated model.

This study focuses exclusively on longitudinal car-following dynamics. However, under low-friction conditions such as snow-covered or icy roads, drivers frequently exhibit lateral oscillations and lane-departure tendencies, and the coupling between longitudinal and lateral control becomes non-negligible. The proposed model does not capture these lateral dynamics, and its predictions should therefore be interpreted as valid only when the vehicle is in a stable car-following state with no imminent lane change or lateral avoidance maneuver. Extending the framework to two-dimensional trajectory prediction, for example by incorporating lateral position, yaw rate, and steering angle into the input feature vector and by adopting a multi-task learning architecture, is an important direction for future research. In addition, the interaction between car-following and lane-changing decisions under snowy conditions warrants dedicated investigation.
